# A novel improved total variation algorithm for the elimination of scratch-type defects in high-voltage cable cross-sections

**DOI:** 10.1371/journal.pone.0300260

**Published:** 2024-04-16

**Authors:** Aihua Yu, Lina Shan, Wen Zhu, Jing Jie, Beiping Hou

**Affiliations:** School of Automation and Electrical Engineering, Zhejiang University of Science and Technology, Hangzhou, Zhejiang Province, China; Newcastle University, UNITED KINGDOM

## Abstract

In the quality inspection process of high-voltage cables, several commonly used indicators include cable length, insulation thickness, and the number of conductors within the core. Among these factors, the count of conductors holds particular significance as a key determinant of cable quality. Machine vision technology has found extensive application in automatically detecting the number of conductors in cross-sectional images of high-voltage cables. However, the presence of scratch-type defects in cut high-voltage cable cross-sections can significantly compromise the precision of conductor count detection. To address this problem, this paper introduces a novel improved total variation (TV) algorithm, marking the first-ever application of the TV algorithm in this domain. Considering the staircase effect, the direct use of the TV algorithm is prone to cause serious loss of image edge information. The proposed algorithm firstly introduces multimodal features to effectively mitigate the staircase effect. While eliminating scratch-type defects, the algorithm endeavors to preserve the original image’s edge information, consequently yielding a noteworthy enhancement in detection accuracy. Furthermore, a dataset was curated, comprising images of cross-sections of high-voltage cables of varying sizes, each displaying an assortment of scratch-type defects. Experimental findings conclusively demonstrate the algorithm’s exceptional efficiency in eradicating diverse scratch-type defects within high-voltage cable cross-sections. The average scratch elimination rate surpasses 90%, with an impressive 96.15% achieved on cable sample 4. A series of conducted ablation experiments in this paper substantiate a significant enhancement in cable image quality. Notably, the Edge Preservation Index (EPI) exhibits an improvement of approximately 20%, resulting in a substantial boost to conductor count detection accuracy, thus effectively enhancing the quality of high-voltage cable production.

## 1 Introduction

High-voltage cables, serving as primary carriers for power transmission, constitute a vital component within the power distribution system. Ensuring the quality of their production carries paramount importance. High-voltage cables typically consist of core conductors, insulation, shielding and protective layers [1]. Consequently, the assessment of high-voltage cable quality typically involves scrutinizing cable length, insulation thickness, and the count of conductors. Among these parameters, the number of conductors present within the cable ranks as one of the most critical indicators of cable quality.

**Fig 1:**
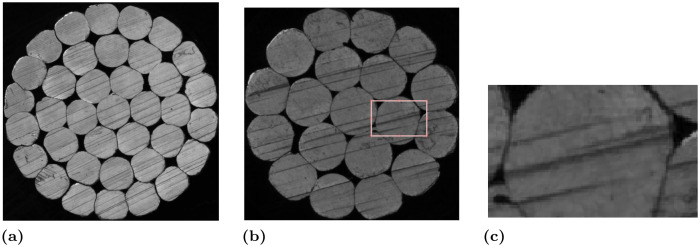
High-voltage cable cross-section images. (**a**) and (**b**) are two different types of high-voltage cable cross-section images, and (**c**) is the local image of (**b**).

Presently, a cost-effective approach involves utilizing machine vision technology to identify the number of conductors in cross-sectional cable images. However, the cable cutting and sampling process often yields numerous scratches, as illustrated in Fig 1. These scratches disrupt the inherent texture structure of the cross-section itself, occasionally leading to the erroneous division of conductors into multiple parts (cf., Fig 1c). Consequently, this phenomenon significantly diminishes the accuracy of conductor count detection. Hence, it is imperative to develop a method capable of effectively eliminating scratch-type defects within the cross-section.

The presence of scratch-type defects on cables can significantly impede the accurate detection of the number of conductors. To address this problem, Dong et al [2] simply mitigated the problem by using a Gabor filter. Recently, Zhang et al [3] designed a novel filter to eliminate scratches on cross-sectional images, which resulted in a significantly higher rate of scratch elimination and gained wide acceptance in the industry. The method mainly focuses on the ultimate removal of scratches by reducing the energy distribution of scratch-type defects in the frequency domain. However, this method exhibits an unstable processing effect in cases of complex scratch distribution and a high number of conductors. Additionally, the process of scratch removal often results in the loss of valuable information. Recognizing these limitations, this paper approaches scratch-type defects as image noise and seeks to eliminate them using denoising methods.

With the advent of Convolutional Neural Networks (CNN) [4], and particularly following the ImageNet 2012 Challenge, deep learning-based defect detection [5, 6], image detection [7–10], image inpainting [11–14] and denoising [15–21] algorithms have gained significant popularity in the field of imaging. Deep learning algorithms can achieve superior performance compared to their predecessors, but they require extensive and consistently distributed data sets for model training. High-voltage cables are expensive, and the amount of sample data available is limited. The generation of scratch-type defects during the cable cutting process is random, resulting in variations in their lengths and depths. Consequently, the available data fails to meet the volume requirements necessary for training deep learning models. Therefore, this paper opts for traditional denoising algorithms to eliminate scratches, as the deep learning approach is not suitable in this context.

**Fig 2:**
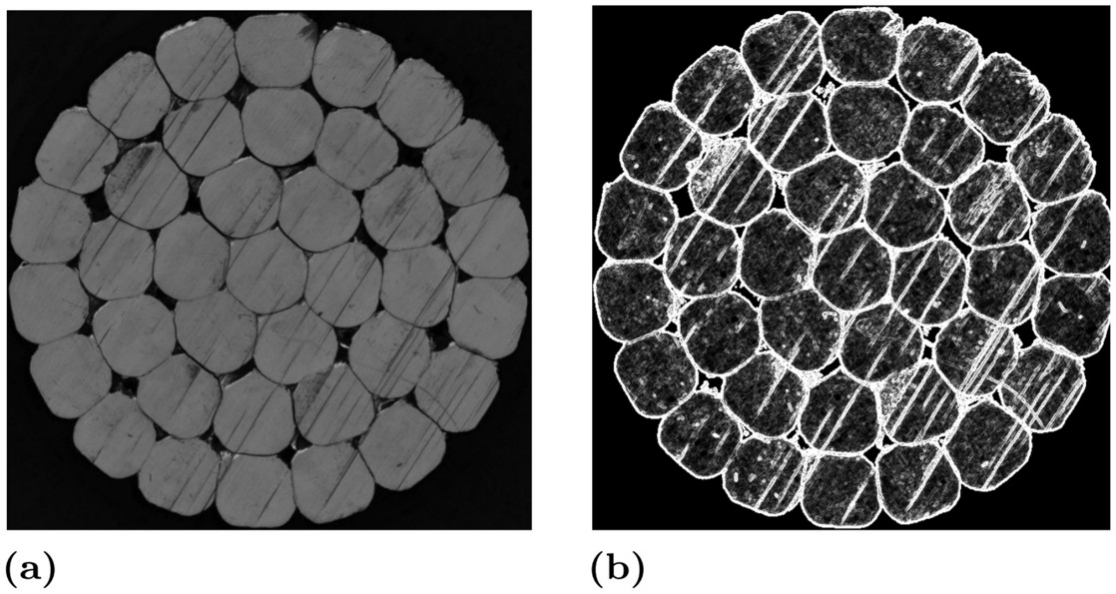
Analysis of the cross-sectional image. (**a**) High-voltage cable Cross-Section image. (**b**) The high-voltage cable cross-section gradient diagram.

There are many traditional denoising methods [22–29]. After analyzing the texture of cable cross-sections, we observed that scratch-free cable cross-sections exhibit minimal gradient changes (Fig 2), except at the image edges. In contrast, scratches introduce significant differences between neighboring pixels, resulting in pronounced gradient changes and an overall increase in the image’s gradient value. As a result, this paper explores the use of the Total Variation (TV) algorithm to reduce the overall image gradient energy as a means of eliminating scratches.

The TV algorithm can effectively remove the image noise and protect the information of the image edges. Rudin et al. [30] originally introduced a regularity parameter in the TV model as part of the concept of partial differential equation denoising, based on the TV of the image gradient. However, this model can lead to the so-called staircase effect. Several methods have been proposed to address this problem, including anisotropic total variation (ATV) [31], total variation methods with regular term combined with hessian matrix [32], non-local total variation (NLTV) [33], iteratively reweighted anisotropic total variation (RwATV) [34], weighted directional total variation (WDTV) [35], and gradient-based directional total variation (BDTV) [36]. While these methods have improved noise suppression noise compared to traditional TV methods, they still lack comprehensive edge and detail preservation. Subsequently, the modifed Barzilai-Borwein method (MBBM) [37] was introduced to improve MR image accuracy, similar to the Fast algorithm for total variation and nuclear norm regularization for dynamic MR image reconstruction (FTVNNR) [38]. Although these techniques effectively address noise issues in traditional TV algorithms, they are primarily applied to medical CT or MR images or used for synthetic noise processing.

Therefore, in the context of eliminating scratch-type defects in high-voltage cable cross-sections, this paper combines multimodal fusion theory with the TV algorithm to develop an improved TV method.

The main contributions of this paper are as follows:

1. A novel improved TV algorithm is proposed, which is effectively eliminates scratches and enhances detection accuracy by employing a multimodal fusion approach.
2. The proposed algorithm achieves an impressive average scratch elimination rate exceeding 90% in high-voltage cable cross-sections. Additionally, there is a notable 30% improvement in the accuracy of conductor count detection, which serves as a more robust quality assurance measure for high-voltage cable production.
3. In this study, a dataset comprising 1,000 high-voltage cable cross-section images is curated. The dataset encompasses five standard sizes of high-voltage cable cross-sections, with 200 images for each size. This dataset is readily available for assessing the performance of various scratch elimination algorithms.

The rest of this paper is organized as follows: Section 2 explains our proposed method in detail, and Section 3 presents the experiments and a discussion of the results.

## 2 Our proposed method

In this paper, an improved TV algorithm is proposed to eliminate many scratch-type defects that often remain in high-voltage cable cross-sections following the cutting process. This algorithm incorporates multimodal features to address conventional challenges associated with the TV algorithm, such as the loss of image information due to the staircase effect. This algorithm first morphological processes the input image to obtain a new modality. The methodology begins with morphological processing of the input image to generate a new modality. Subsequently, the fused images from multiple modalities are computed and utilized as input for the TV algorithm, ultimately resulting in the effective elimination of scratch-type defects in high-voltage cables. In the following sections, we provide a detailed exploration of three key aspects: the TV algorithm, multimodal fusion, and the improved TV algorithm.

### 2.1 The total variation algorithm

In this subsection, we delve into the theoretical underpinnings of the TV algorithm. Rudin et al. [30] introduced the TV algorithm as a comprehensive model for image denoising through the application of partial differential equations. The core concept of this model revolves around the reduction of the total sum of gradient integrals within the pixel domain of an image. The model permits the presence of sharp discontinuous points in the image, however, it is susceptible to staircase effects that result in the disappearance of texture and blurring of the image [39].

A noisy image is typically composed of original image information and noise, and the definitions are as follows:

$$\begin{array}{c} I(x,y)=h(x,y)+n(x,y). \end{array}$$ (1)

where **n**(*x*, *y*) is noise.

The TV algorithm is described as follows:

$$\begin{array}{c} T[h(x,y)]=\underset{\Omega }{\;\int \int \;}|\nabla h(x,y)|dxdy, \end{array}$$ (2)

where *Ω* is the pixel domain of the entire image, $|\nabla h|=\sqrt{h_{x}^{2}+h_{y}^{2}}$, $h_{x}=\frac{\partial h}{\partial x}$, $h_{y}=\frac{\partial h}{\partial y}$.

In the context of denoising, the TV algorithm must be transformed into an energy generalized function before being applied.

$$\begin{array}{c} \begin{array}{cc} J[h(x,y)] & =T[h(x,y)]+\frac{\lambda }{2}\underset{\Omega }{\;\int \int \;}{\left[h(x,y)-a(x,y)\right]}^{2}dxdy\\ & =\underset{\Omega }{\;\int \int \;}|\nabla h(x,y)|dxdy+\frac{\lambda }{2}\underset{\Omega }{\;\int \int \;}{\left[h(x,y)-a(x,y)\right]}^{2}dxdy, \end{array} \end{array}$$ (3)

where **a** is a noisy image, λ represents the weight parameter in this section.

Eq (3) contains the TV regularization term for smoothing the image, and the fidelity term for preserving the original image features and reducing image loss. The two terms are balanced by the λ parameter. By utilizing variational lemmas and Euler-Lagrange equation, Eq (3) is transformed into an equation for an extreme value (Eq (4)).

$$\begin{array}{c} -\nabla \left(\frac{\nabla h}{\left|\nabla h\right|}\right)+\lambda \left(h-a\right)=0, \end{array}$$ (4)

where ∇ is the symbol for the total differential operation. $\frac{1}{\left|\nabla h\right|}$ is the coefficient of diffusion.

At the edges of the image, the value of |∇**h**| is larger, the diffusion coefficient is smaller, resulting in weaker diffusion in the direction of the edges. This helps to preserve the details of the edges. In the region where smoothing occurs, the value of |∇**h**| is smaller, the diffusion coefficient is larger, which increases the diffusion ability for more effective to eliminate the internal noise in the image.

### 2.2 Multimodal fusion

Each source or image form is referred to as a modality. These different modalities are represented distinctively, containing varying image information, resulting in intersections and complementarity of information. Consequently, the enrichment of image features can be achieved through the fusion of information from multiple modalities.

To better preserve the edge information within images, we propose a specialized multimodal fusion method in this paper. This method encompasses diverse treatments applied to the same image, such as sparsizing, filtering and morphological, to generate distinct modalities. After careful analysis and comparison of these methods, we have opted for the morphological processing approach.

In morphology, dilation operation expands regions, extending object edges outward to fill small cracks or voids. These operations can also thicken or connect objects. Conversely, erosion operations reduce the area by retracting object edges inward, thus eliminating noise and fine objects.

In this paper, our approach begins by dilating the image, followed by eroding the inflated image. this process eliminates small gaps between objects and mends breaks in contour lines while maintaining the original shape and size of the objects. Although resembling the close operation in morphology, we employ different structural elements here. Structural elements are used to define neighborhood relations in morphological operations, which can be binary matrices of arbitrary shape and size. This paper selects ellipsoidal structure elements of varying sizes based on the qualities of scratch-type defects. Definitions are as follows:

$$\begin{array}{c} \begin{array}{cc} I_{B} & =M(I_{A}),\\ M(I_{A}) & =\left(I_{A}⊕S_{1}\right)⊖S_{2}. \end{array} \end{array}$$ (5)

where **I**_*A*_ is the input image, **I**_*B*_ is the morphologically processed image. *S*_1_ and *S*_2_ are the structural elements used in the dilation and erosion processes, respectively. ⊕ and – are symbols for dilation and erosion operation.

**Fig 3:**
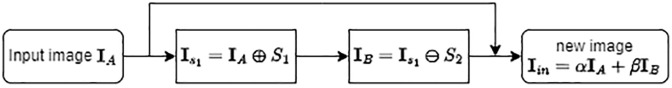
Multimodal fusion flowchart.

Then the images of different modalities are fused to get a new image **I**_*in*_. The flowchart is shown in Fig 3.

$$\begin{array}{c} \begin{array}{cc} I_{in} & =\alpha I_{A}+\beta I_{B} \end{array} \end{array}$$ (6)

where *α* and *β* are the different modality weights, 0 ≤ *α* ≤ 1, 0 ≤ *β* ≤ 1, and *α* + *β* = 1.

### 2.3 Improved total variation algorithm

Considering the characteristics of the TV algorithm and the analysis of scratch-type defects, it is evident that the TV algorithm is well-suited for elimination of scratch-type defects. Nevertheless, the conventional TV algorithm often leads to a significant blurring of graphic edges due to the pronounced staircase effect. In response to this challenge, our paper introduces an improved TV algorithm that augments image information via multimodal fusion. This approach mitigates the impact of the staircase effect and enhances the preservation of graphic edges more effectively.

The algorithm first calculates a multimodal fusion image **I**_*in*_ using Eqs (5) and (6), and uses **I**_*in*_ as input for Eq (7). The initial value for the output image **I** is set to the original image **I**_*A*_, and finally, iterative updates are performed to obtain the output image I* with scratch-type defects eliminated. The Eq (7) is as follows:

$$\begin{array}{c} \begin{array}{cc} I^{*} & =\text{arg}\;\underset{I}{\text{min}}J[I(x,y)]\\ & =\text{arg}\;\underset{I}{\text{min}}\frac{1}{2}\underset{\Omega }{\;\int \int \;}{\left[I\left(x,y\right)-I_{in}\left(x,y\right)\right]}^{2}dxdy+\lambda \underset{\Omega }{\;\int \int \;}|\nabla I(x,y)|dxdy \end{array} \end{array}$$ (7)

where *Ω* represents the pixel domain of the entire image, and λ is the regularization parameter. The first term on the right-hand side is the fidelity term, and the second term contains the multimodality TV regularization term.

Given that

$$\begin{array}{c} \left\{ \begin{array}{cc} J[I(x,y)] & =\underset{\Omega }{\;\int \int \;}F[x,y,I\left(x,y\right),\frac{\partial I}{\partial x},\frac{\partial I}{\partial y}]dxdy,\\ \left|\nabla I\right| & =\sqrt{{\left(\frac{\partial I}{\partial x}\right)}^{2}+{\left(\frac{\partial I}{\partial y}\right)}^{2}},\\ \nabla I & =\frac{\partial I}{\partial x}+\frac{\partial I}{\partial y},\\ \;I_{x} & =\frac{\partial I}{\partial x},\\ \;I_{y} & =\frac{\partial I}{\partial y}. \end{array}\right. \end{array}$$ (8)

the function *F* is defined as follows:

$$\begin{array}{c} \begin{array}{cc} F & =\lambda |\nabla I|+\frac{1}{2}{\left[I\left(x,y\right)-I_{in}\left(x,y\right)\right]}^{2}\\ & =\lambda \sqrt{{\left(\frac{\partial I}{\partial x}\right)}^{2}+{\left(\frac{\partial I}{\partial y}\right)}^{2}}+\frac{1}{2}{\left[I-I_{in}\right]}^{2} \end{array} \end{array}$$ (9)

where *F* serves as a shorthand for $F[x,y,I\left(x,y\right),\frac{\partial I}{\partial x},\frac{\partial I}{\partial y}]$, and the same is applicable to **I** and **I**_*in*_.

To find the minimum value of function *F*, it is necessary to satisfy the Euler-Lagrange equation. The Euler-Lagrange equation (Eq (11)) for function *F* can be obtained by using Eq (10).

$$\begin{array}{c} \left\{ \begin{array}{cc} \frac{\partial F}{\partial I} & =\frac{\partial }{\partial x}\left(\frac{\partial F}{\partial I_{x}}\right)+\frac{\partial }{\partial y}\left(\frac{\partial F}{\partial I_{y}}\right),\\ \frac{\partial F}{\partial I} & =I\left(x,y\right)-I_{in}\left(x,y\right),\\ \frac{\partial F}{\partial I_{x}} & =\lambda \frac{\frac{\partial I}{\partial x}}{\sqrt{{\left(\frac{\partial I}{\partial x}\right)}^{2}+{\left(\frac{\partial I}{\partial y}\right)}^{2}}}=\lambda \frac{\frac{\partial I}{\partial x}}{\left|\nabla I\right|},\\ \frac{\partial F}{\partial I_{y}} & =\lambda \frac{\frac{\partial I}{\partial y}}{\sqrt{{\left(\frac{\partial I}{\partial x}\right)}^{2}+{\left(\frac{\partial I}{\partial y}\right)}^{2}}}=\lambda \frac{\frac{\partial I}{\partial y}}{\left|\nabla I\right|}. \end{array}\right. \end{array}$$ (10)

$$\begin{array}{c} \begin{array}{c} \lambda \left[\frac{\partial }{\partial x}\left(\frac{\frac{\partial I}{\partial x}}{\left|\nabla I\right|}\right)+\frac{\partial }{\partial y}\left(\frac{\frac{\partial I}{\partial y}}{\left|\nabla I\right|}\right)\right]=I-I_{in} \end{array} \end{array}$$ (11)

Therefore, the optimised output image **I**_*k*+1_ for the *k*-th iteration can be obtained from Eq (12).

$$\begin{array}{c} \begin{array}{c} I_{k+1}=\lambda \left[\nabla \left(\frac{\nabla I_{k}}{\left|\nabla I_{k}\right|}\right)\right]+I_{in} \end{array} \end{array}$$ (12)

where $\left|\nabla I_{k}\right|=\sqrt{{\left|\nabla I_{k}\right|}^{2}+\varepsilon }$, *ε* > 0. This is to avoid the value of |∇**I**_*k*_| being zero in the flat region of the image in the actual calculation, which makes $\nabla (\frac{\nabla I_{k}}{\left|\nabla I_{k}\right|})$ meaningless.

The steps to implement the improved TV algorithm 1 are shown below:

**Algorithm 1:** Improved TV algorithm

**Input:** the original image $I_{A}\in R^{H\times W}$, *α* ∈ (0, 1), *β* ∈ (0, 1), λ = 0.25, *N* = 175;

**Output:** $I_{k+1}\in R^{H\times W}$ ;

**1** $I_{B}=M\left(I_{A}\right)$ ;

**2 I**_*in*_ = *α***I**_*A*_ + *β* **I**_*B*_;

**3** *k* ← 1;

**4 while** *k* + 1 ≤ *N* **do**

**5** **if** *k* = 1 **then**

**6**  **I**_*k*_ ← **I**_*in*_;

**7**  **end**

**8**  $\nabla I_{k}=\frac{\partial I_{k}}{\partial x}+\frac{\partial I_{k}}{\partial y}$;

**9**  $|\nabla I_{k}|=\sqrt{{\left(\frac{\partial I_{k}}{\partial x}\right)}^{2}+{\left(\frac{\partial I_{k}}{\partial y}\right)}^{2}}$;

**10** $I_{k+1}=\lambda \left[\nabla \left(\frac{\nabla I_{k}}{\left|\nabla I_{k}\right|}\right)\right]+I_{in}$;

**11** *k* ← *k* + 1;

**12 end**

**13 I**_*k*+1_

## 3 Experiments

**Fig 4:**
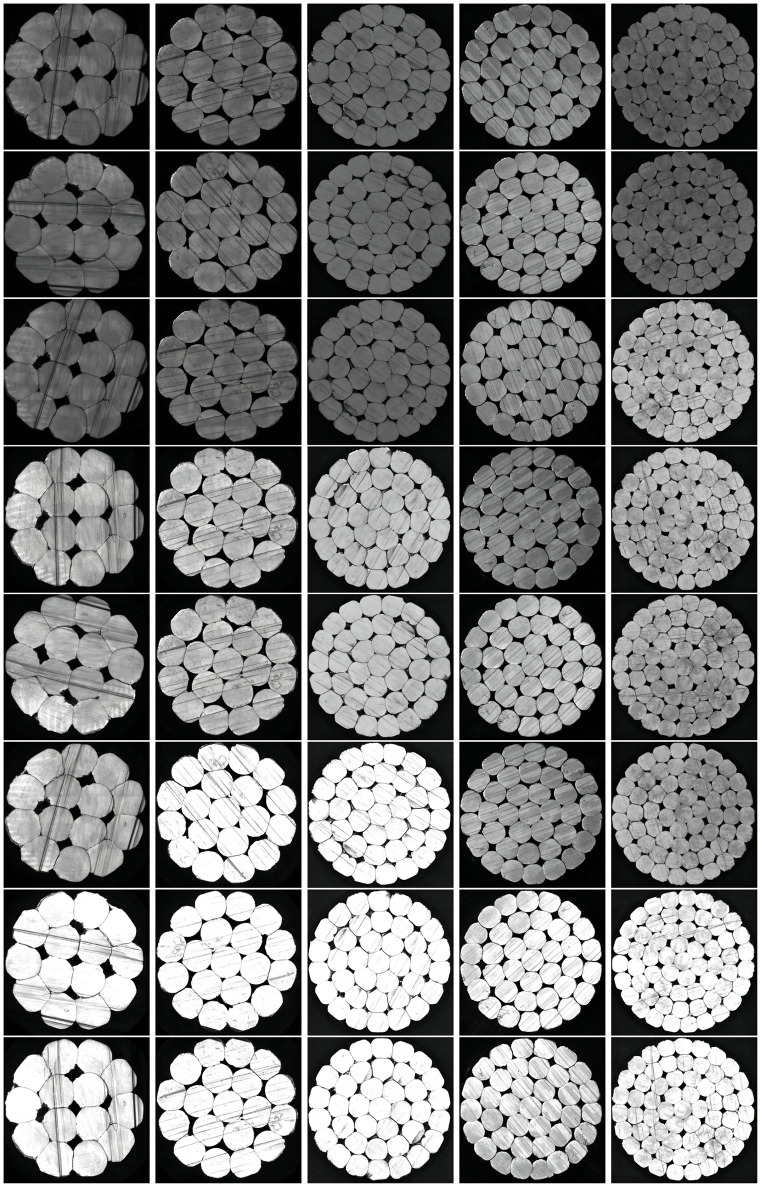
Some of the images of this dataset.

In this study, an image acquisition platform for capturing high-voltage cable cross-sections was constructed using components such as an industrial camera, optical lens, ring light source, and tablet PC. A comprehensive dataset was created experimental utilizing this platform. This dataset consists of a total of 1,000 images. This dataset encompasses five standard sizes of high-voltage cable cross-sections, with 200 images for each size. A selection of these dataset images is displayed in Fig 4. The number of roots of high-voltage cables used in this experiment are mainly 14, 19, 37, 37 and 60, while allowing the input of cable images of any size. The cable image sizes used in this paper are around 330 × 330, 420 × 420, 600 × 600, 680 × 680 and 770 × 770.

In the following experiments, the improved TV algorithm proposed in this paper (Method A) is compared and analysed with the improved frequency domain filtering algorithm proposed in [3] (Method B) as well as the traditional TV algorithm (Method C), by utilizing our self-produced dataset. All experiments were executed on a PC with Intel(R) Core(TM) i5–8250U CPU @ 1.60 GHz, complemented by 8.00 GB of RAM, and MATLAB R2020a served as the software platform. It is important to note that the parameters for our proposed method have been meticulously optimized in accordance with industry expertise, with specific values set as follows: *α* = 0.676, *β* = 0.324, and λ = 0.25.

### 3.1 Experiment on the eliminative effect of scratch-type defects in the cross-section of high-voltage cables

To assess the performance of our proposed method (Method A), a comparative analysis between Method A and Method B on our self-constructed dataset is conducted. The evaluation metric employed in the experiment is the average elimination rate, which is defined as follows:

$$\begin{array}{c} \begin{array}{c} v_{k}≜\frac{1}{N_{k}}\sum_{i=1}^{N_{k}}\frac{m_{k,i}}{M_{k,i}}\times 100\%,\;k=1,2,\cdots ,K \end{array} \end{array}$$ (13)

where *v*_*k*_ denotes the average elimination rate of scratches of the high-voltage cable of the *k*-th size, *N*_*k*_ denotes the number of images of the cable of the *k*-th size, *M*_*k*,*i*_ denotes the total number of scratches contained in the *i*-th image of the cable of the *k*-th size, *m*_*k*,*i*_ denotes the number of scratches eliminated from the *i*-th image of the cable of the *k*-th size, and *K* denotes the total number of sizes of the cable. In this experiment, *K* = 5, *N*_1,2,…,*K*_ = 200.

**Fig 5:**
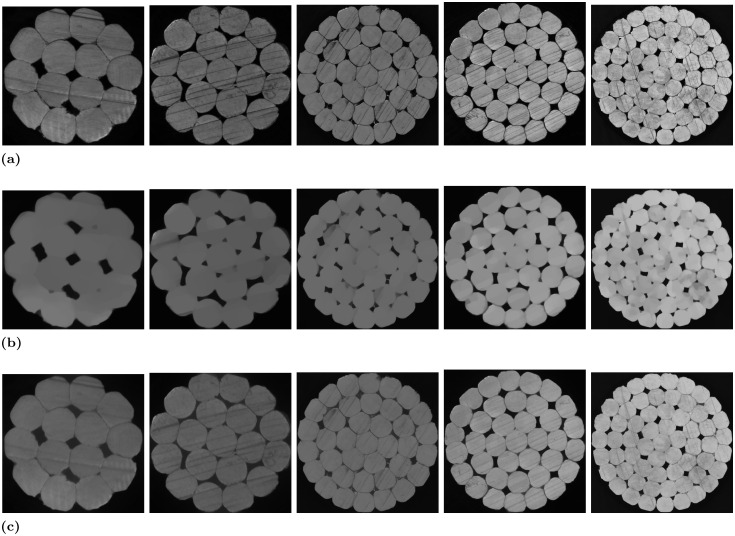
Images of the effect of eliminating scratch-type defects in the cross-section of high-voltage cables. From left to right, Sample 1 to Sample 5. Fig 5a are original cross-sections of high-voltage cables with scratch-type defects. Fig 5b are the elimination effect images of the improved TV algorithm (Method A) proposed in this paper. Fig 5c are images of the elimination effect of the improved frequency domain filtering method (Method B) proposed in [3].

Some of the experimental visualisations are displayed in Fig 5, and Table 1 shows the comprehensive results of the experiment.

**Table 1:** Average elimination rates of scratch-type defects for Method A and Method B on different sizes of high-voltage cables.

Sample	Method A (our)	Method B
Sample 1	93.17%	24.72%
Sample 2	95.70%	29.38%
Sample 3	95.60%	40.50%
Sample 4	96.15%	34.84%
Sample 5	92.95%	49.82%

Upon a thorough examination of the experimental outcomes presented in Fig 5, it is evident that Method B proves proficient at eliminating most shallow scratches. However, it struggles with deeper scratches. Furthermore, the efficacy of this method diminishes when confronted with high-voltage cables featuring a substantial number of conductors and a complex distribution of scratch-type defects. Conversely, as illustrated in the Fig 5b, Method A successfully addresses these issues by efficiently eliminating the majority of the scratch-type defects.

Table 1 clearly illustrates the elimination rates achieved by the different methods. Method B only approaches 50% in specific cable specifications, while the proposed Method A consistently achieves elimination rates exceeding 90% across all specifications.

The primary factor contributing to the superiority of our proposed method over Method B lies in the approach used. Method B relies on a filtering technique to reduce the energy distribution of scratch-type defects in the frequency domain, ultimately aiming for their removal. However, due to variations in the depth and length of the scratches, their distribution in the frequency domain is not concentrated. Consequently, the filter struggles to effectively eliminate these scratches. In contrast, our method significantly enhances edge information through multimodal fusion, after which the scratch-type defects will be well removed by the TV algorithm.

### 3.2 Conductor number detection experiment

To validate the enhanced performance of Method A in detecting the number of conductors within high-voltage cables, we consistently employed the Hough circle detection algorithm for conductor counting across our self-constructed dataset. The ensuing experiments were meticulously conducted as follows:

1. After processing the original image using Method A, the output image was subjected to Hough circle detection;
2. After processing the original image using Method B, perform Hough circle detection on the output image;
3. Directly perform Hough circle detection on the original image.

The evaluation metric used in the experiment is the average detection rate of the number of conductors, which is defined as follows:

$$\begin{array}{c} \begin{array}{c} v_{d,k}≜\frac{1}{N_{k}}\sum_{i=1}^{N_{k}}\frac{d_{k,i}}{D_{k,i}}\times 100\%,\;k=1,2,\cdots ,K \end{array} \end{array}$$ (14)

where *v*_*d*,*k*_ denotes the average detection rate of the number of conductors of the *k*-th size, *N*_*k*_ denotes the number of images of the cable of the *k*-th size, *D*_*k*,*i*_ denotes the total number of conductors in the *i*-th image of the cable of the *k*-th size, *d*_*k*,*i*_ denotes the number of conductors detected from the *i*-th image of the cable of the *k*-th size, and *K* denotes the total number of sizes of the cable. In this experiment, *K* = 5, *N*_1,2,…,*K*_ = 200.

**Fig 6:**
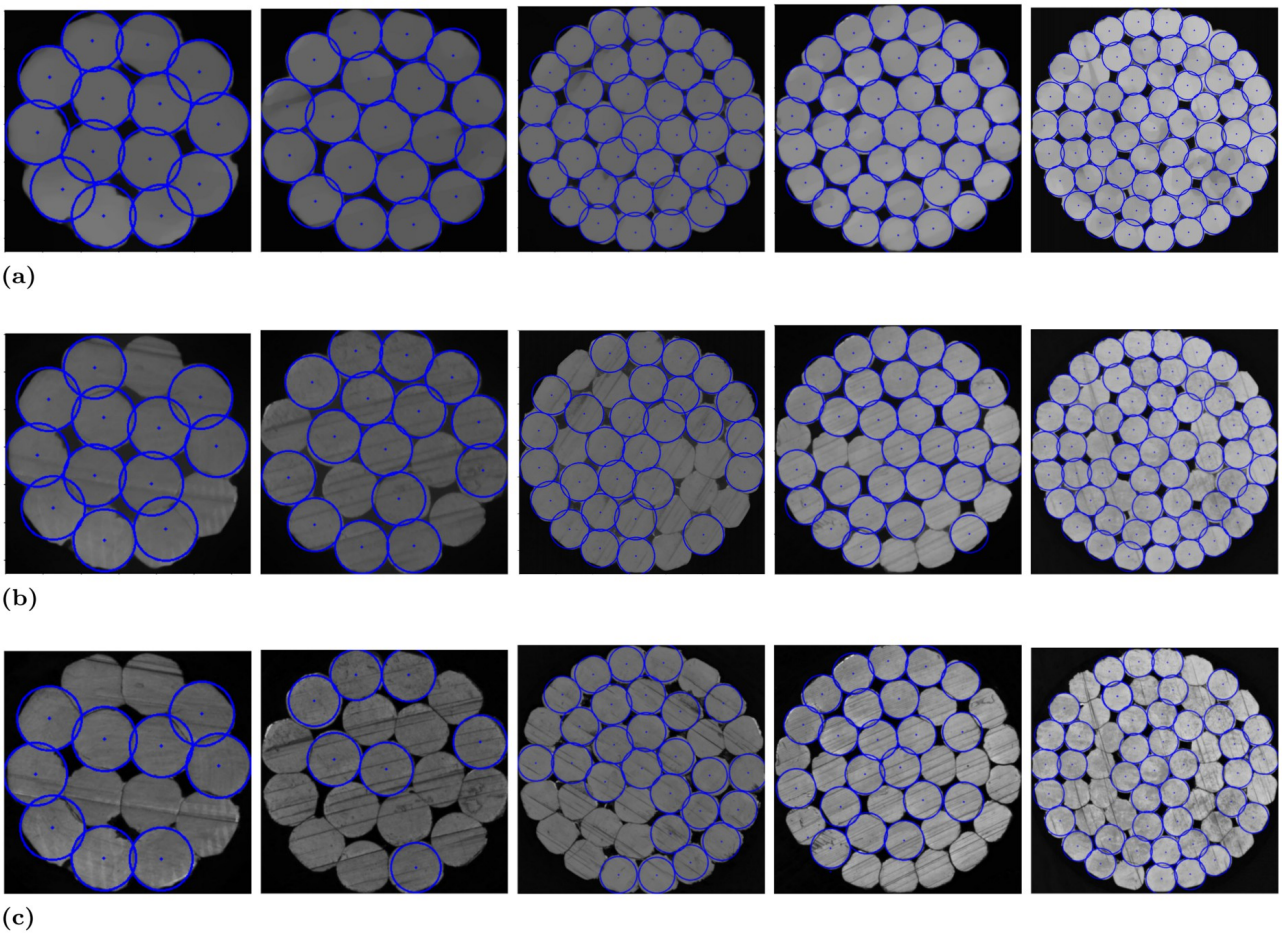
Conductor number detection effect images. Fig 6a shows the detection effect after using method A (our), Fig 6b shows the detection effect after using method B, and Fig 6c shows the effect of direct detection.

Some of the experimental visualisations are displayed in Fig 6, while Table 2 shows the comprehensive results of the experiment.

**Table 2:** Average detection rate of the number of conductors for different methods.

Sample	Method A (our)	Method B	The direct detection
Sample 1	95.89%	85.18%	64.54%
Sample 2	96.92%	79.11%	58.39%
Sample 3	96.34%	76.08%	70.09%
Sample 4	95.73%	78.78%	57.36%
Sample 5	97.17%	89.81%	68.39%

By observing Fig 6, it becomes evident that the direct detection yields subpar results, primarily due to the presence of deep scratches that erroneously split a single conductor into two, thereby adversely affecting the accuracy of conductor number detection. The limitation of Method B lies in its partial elimination of scratches, focusing primarily on the shallower ones. Consequently, the presence of uneliminated scratches in Method B disrupts the accurate detection of the actual number of conductors.

In contrast, Method A stands out by effectively eliminating the vast majority of scratches while preserving essential image edge information. This substantial improvement sets Method A apart from the previous two methods, markedly enhancing the precision and quality of conductor number detection.

Table 2 clearly illustrates that our method (Method A) leads to a substantial enhancement in conductor number detection performance, notably increasing the average detection rate by approximately 30%. When compared to Method B, our method (Method A) achieves a noteworthy 1.5-fold improvement in the average detection rate.

### 3.3 Ablation experiment

To showcase the effectiveness of the multimodal fusion method introduced in this paper’s algorithm, this section presents ablation experiments. The original method is contrasted with Method C, which excludes the multimodal fusion. Evaluation criteria for assessing scratch-type defect elimination encompass Peak signal-to-noise ratio (PSNR), Structural Similarity (SSIM) [40], average elimination rate, and running time. PSNR and SSIM are defined by Eqs (15) and (16), respectively.

$$\begin{array}{c} \begin{array}{cc} PSNR & =10\;{\text{log}}_{10}\left(\frac{MAX_{\hat{I}}^{2}}{MSE}\right)\\ MSE & =\frac{1}{mn}\sum_{i=0}^{m-1}\sum_{j=0}^{n-1}{∥\hat{I}\left(i,j\right)-\overset{ˇ}{I}\left(i,j\right)∥}^{2} \end{array} \end{array}$$ (15)

where *m* and *n* are the image sizes, $\hat{I}$ is the image after removal of scratch-type defects, $\overset{ˇ}{I}$ is the image with scratch-type defects, $MAX_{\hat{I}}$ is the maximum possible pixel value in image $\hat{I}$.

$$\begin{array}{c} \begin{array}{c} SSIM\left(x,y\right)={\left[l\left(\hat{I},\overset{ˇ}{I}\right)\right]}^{q_{1}}\cdot {\left[c\left(\hat{I},\overset{ˇ}{I}\right)\right]}^{q_{2}}\cdot {\left[s\left(\hat{I},\overset{ˇ}{I}\right)\right]}^{q_{3}} \end{array} \end{array}$$ (16)

$$\begin{array}{c} \left\{ \begin{array}{c} l\left(x,y\right)=\frac{2\mu_{\hat{I}}\mu_{\overset{ˇ}{I}}+C_{1}}{\mu_{\hat{I}}^{2}+\mu_{\overset{ˇ}{I}}^{2}+C_{1}},\\ c\left(x,y\right)=\frac{2\sigma_{\hat{I}\overset{ˇ}{I}}+C_{2}}{\sigma_{\hat{I}}^{2}+\sigma_{\overset{ˇ}{I}}^{2}+C_{2}},\\ s\left(x,y\right)=\frac{\sigma_{\hat{I}\overset{ˇ}{I}}+C_{3}}{\sigma_{\hat{I}}\sigma_{\overset{ˇ}{I}}+C_{3}} \end{array}\right. \end{array}$$

where $\mu_{\hat{I}}$, $\mu_{\overset{ˇ}{I}}$, $\sigma_{\hat{I}}$, $\sigma_{\overset{ˇ}{I}}$, and $\sigma_{\hat{I}\overset{ˇ}{I}}$ are the means, standard deviations, and cross-covariance for images $\hat{I}$, $\overset{ˇ}{I}$. *q*_1_, *q*_2_ and *q*_3_ are used to adjust the weights of the three modules, usually 1. *C*_1_, *C*_2_ and *C*_3_ are constants that avoid a denominator of zero.

**Fig 7:**
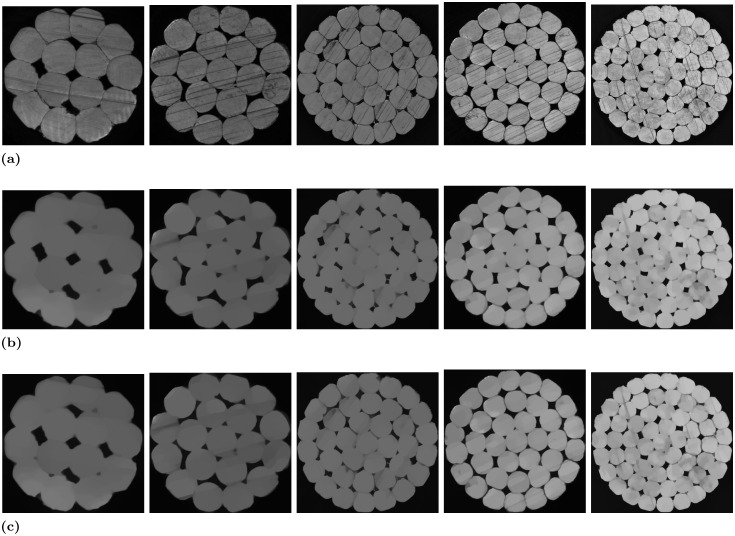
Images of the effect of eliminating scratch-type defects in the cross-section of high-voltage cables. From left to right, Sample 1 to Sample 5. Fig 7a are cross-sections of high-voltage cables with scratch-type defects. Fig 7b are the eliminative effect images of the improved TV algorithm (Method A) proposed in this paper. Fig 7c are eliminative effect images of the for traditional TV algorithm (Method C).

As illustrated in the visual comparison results of the ablation experiments displayed in Fig 7, both methods demonstrate the capacity to eliminate the majority of scratches when utilizing the same experimental data and parameters. Nonetheless, Method C falls short in fully eliminating certain scratches. A detailed analysis in Table 3 highlights the superior performance of Method A, as evidenced by markedly higher PSNR and SSIM values compared to Method C. Notably, the PSNR consistently exceeds 0.75 for Method A, indicating its ability to better preserve valuable image information while effectively eliminating scratch-type defects. While the improvement in SSIM may not be as pronounced, the method proposed in this paper significantly enhances the average elimination rate, achieving an impressive 96.15% on cable sample 4. It’s worth noting that, despite Method A involving an additional step of multimodal fusion compared to Method C, the overall running speed experiences minimal reduction.

**Table 3:** Comparative data of two methods for eliminating scratch-type defects.

Sample	Experimental methods	PSNR/dB	SSIM	Average elimination rate	Running time/s
Sample 1	Method A	**31.748**	**0.841**	**93.17%**	4.281
	Method C	30.120	0.801	87.33%	3.969
Sample 2	Method A	**30.227**	**0.804**	**95.70%**	7.109
	Method C	28.364	0.736	89.18%	6.547
Sample 3	Method A	**31.320**	**0.876**	**95.60%**	19.828
	Method C	29.302	0.833	82.14%	17.031
Sample 4	Method A	**28.628**	**0.767**	**96.15%**	17.500
	Method C	26.460	0.691	86.14%	13.156
Sample 5	Method A	**28.136**	**0.772**	**92.95%**	31.625
	Method C	26.230	0.700	76.16%	23.875

To further emphasize the advantages offered by the methodology proposed in this paper, we have computed the improvement rates for the Edge Preservation Index (EPI) [41], PSNR, and SSIM. The formula for the improvement rate (17) is as follows.

$$\begin{array}{c} R_{k}≜\frac{M_{A,k}-M_{C,k}}{M_{A,k}}\times 100\% \end{array}$$ (17)

where *R*_*k*_ is the improvement rate of k-category metrics, *M*_*A*,*k*_ and *M*_*C*,*k*_ are the values of k-category image quality metrics after using Method A and Method C, respectively. *k* is EPI, PSNR, and SSIM.

Table 4 offers clear evidence of the substantial enhancement in image quality achieved by the methodology presented in this paper after the removal of scratch-type defects. Across all samples, the Edge Preservation Index (EPI) metric exhibits an improvement of approximately 20%, signifying superior preservation of image edge information. Consequently, the processed images are rendered more stable and suitable for downstream tasks.

**Table 4:** Comparative data on the improvement rate of two methods for eliminating scratch-type defects.

R_*k*_	Sample 1	Sample 2	Sample 3	Sample 4	Sample 5
*R*_*EPI*_	25.97%	27.52%	20.78%	23.49%	19.33%
*R*_*PSNR*_	5.41%	6.57%	6.89%	8.19%	7.27%
*R*_*SSIM*_	4.99%	9.24%	5.16%	11.00%	10.29%

In conclusion, the introduction of the multimodal fusion technique in this paper significantly elevates the TV algorithm’s performance in the realm of scratch removal. This improvement positions our approach as a valuable contribution with potential applications in the cable quality inspection industry, promising ease of generalization.

## 4 Conclusions

The cutting process of high-voltage cables leaves many scratches on the cross-section, which affect the detection of the number of conductors. This paper proposes an improved TV algorithm to eliminate these scratch-type defects. The algorithm first performs morphological processing on the input image to obtain a new modality, and then the fused image of multiple modalities is calculated and the fused image is used as an input to the TV algorithm, which ultimately achieves the elimination of scratch-type defects in high-voltage cables.

Based on our experimental results, it can be concluded that the method proposed in this paper achieves an elimination rate of over 90% for scratches. This leads to an average detection rate of over 95% of the number of conductors, greatly surpassing the current mainstream methods utilized in the industry. As our algorithm introduces multimodal features, it can better enhance the image quality and alleviate the edge loss problem caused by the staircase effect, which leads to a more significant improvement in PSNR, SSIM, and EPI, and makes the processing more stable.

Therefore, the algorithm presented in this study can greatly enhance the precision of detecting the quantity of conductors and the quality of manufacturing high-voltage cables. However, the method discussed in this paper has only been successful in laboratory conditions, and the costly cameras and processors utilized do not currently permit large-scale deployment in practical production. Therefore, our future research direction is to make our algorithm run stably under cheaper equipment and more severe conditions, so as to realize the large-scale promotion of its use in the industry.

## Supporting information

S1 Data (ZIP)
