# Supplementary figures and images for: A novel improved total variation algorithm for the elimination of scratch-type defects in high-voltage cable cross-sections
Source: PLoS One. 2024 Apr 16;19(4):e0300260. doi: 10.1371/journal.pone.0300260 (PMC11020849; doi:10.1371/journal.pone.0300260)

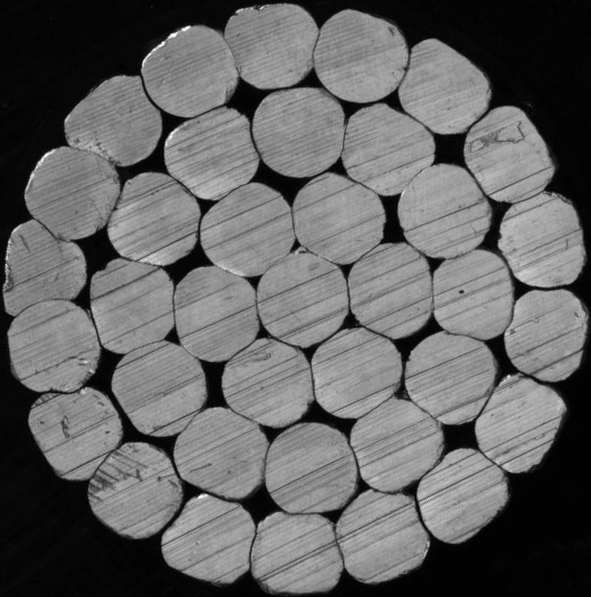

Supplement: S1 Data — (ZIP) [file pone.0300260.s001.zip › Paper_data/Fig_1/a.png]

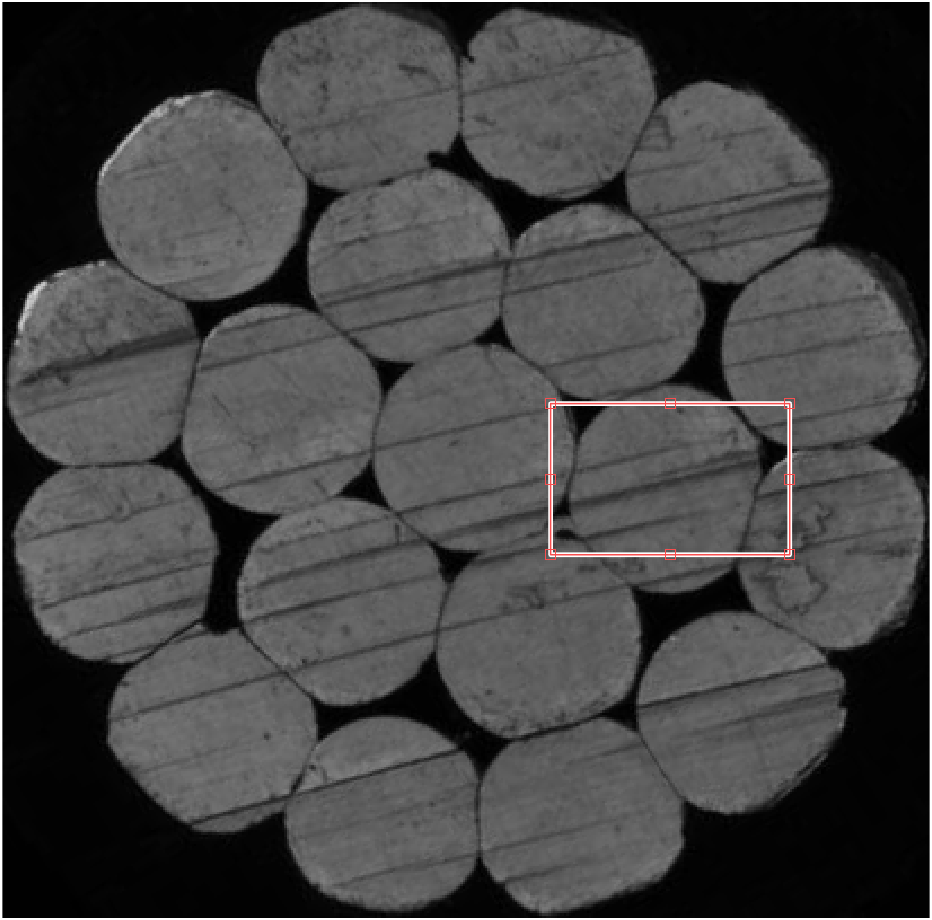

Supplement: S1 Data — (ZIP) [file pone.0300260.s001.zip › Paper_data/Fig_1/b.png]

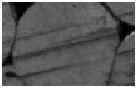

Supplement: S1 Data — (ZIP) [file pone.0300260.s001.zip › Paper_data/Fig_1/c.png]

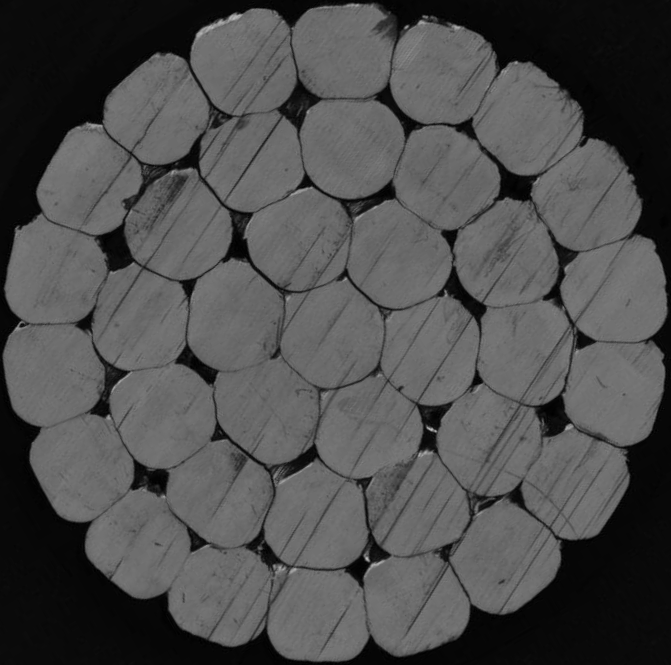

Supplement: S1 Data — (ZIP) [file pone.0300260.s001.zip › Paper_data/Fig_2/a.png]

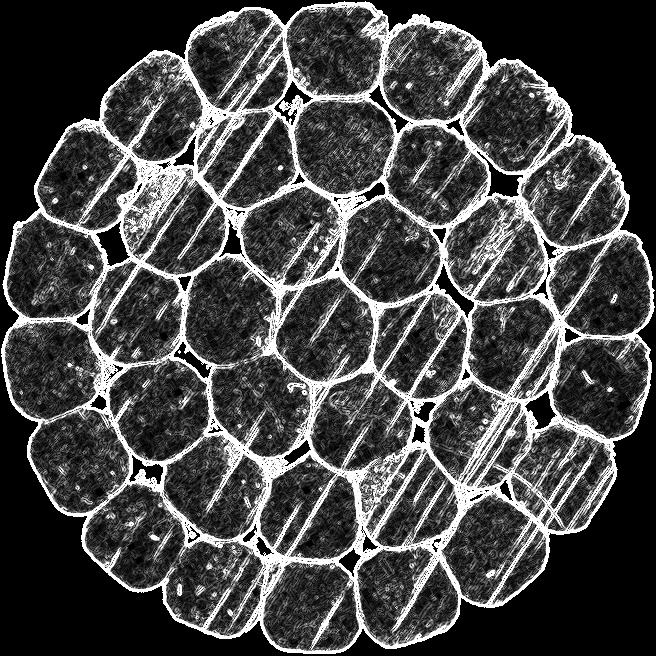

Supplement: S1 Data — (ZIP) [file pone.0300260.s001.zip › Paper_data/Fig_2/b.png]

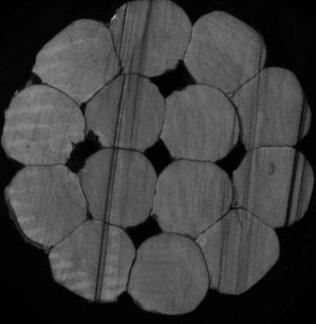

Supplement: S1 Data — (ZIP) [file pone.0300260.s001.zip › Paper_data/Fig_3/1_column/1.jpg]

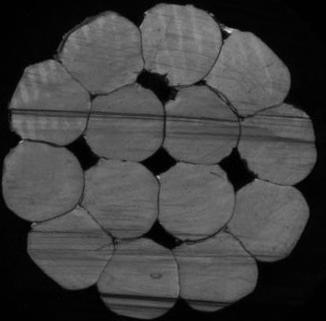

Supplement: S1 Data — (ZIP) [file pone.0300260.s001.zip › Paper_data/Fig_3/1_column/2.jpg]

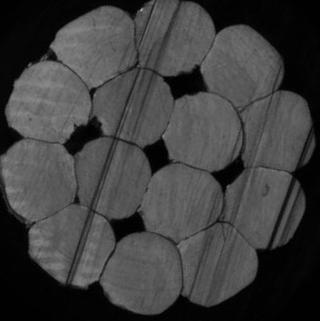

Supplement: S1 Data — (ZIP) [file pone.0300260.s001.zip › Paper_data/Fig_3/1_column/3.jpg]

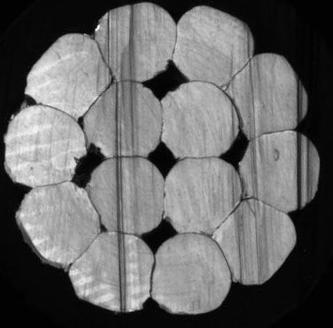

Supplement: S1 Data — (ZIP) [file pone.0300260.s001.zip › Paper_data/Fig_3/1_column/4.jpg]

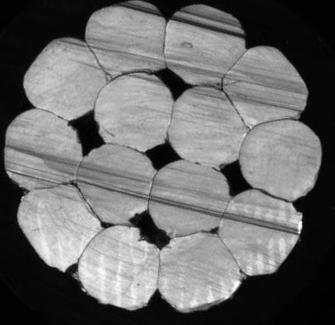

Supplement: S1 Data — (ZIP) [file pone.0300260.s001.zip › Paper_data/Fig_3/1_column/5.jpg]

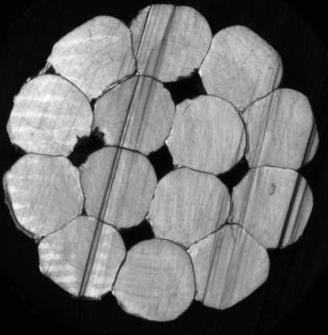

Supplement: S1 Data — (ZIP) [file pone.0300260.s001.zip › Paper_data/Fig_3/1_column/6.jpg]

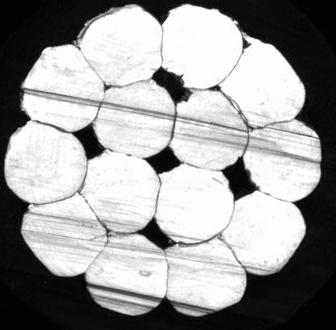

Supplement: S1 Data — (ZIP) [file pone.0300260.s001.zip › Paper_data/Fig_3/1_column/7.jpg]

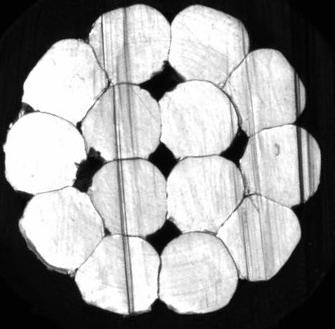

Supplement: S1 Data — (ZIP) [file pone.0300260.s001.zip › Paper_data/Fig_3/1_column/8.jpg]

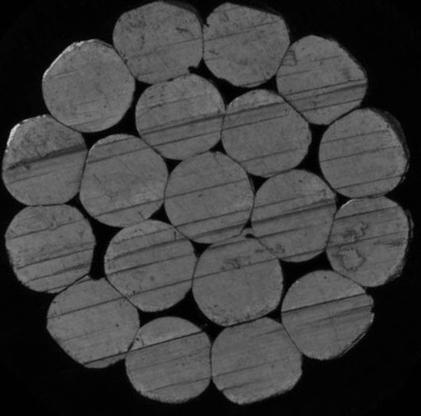

Supplement: S1 Data — (ZIP) [file pone.0300260.s001.zip › Paper_data/Fig_3/2_column/1.jpg]

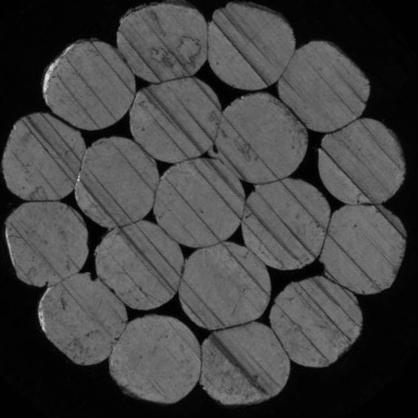

Supplement: S1 Data — (ZIP) [file pone.0300260.s001.zip › Paper_data/Fig_3/2_column/2.jpg]

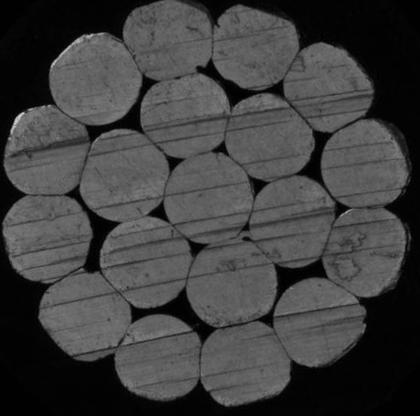

Supplement: S1 Data — (ZIP) [file pone.0300260.s001.zip › Paper_data/Fig_3/2_column/3.jpg]

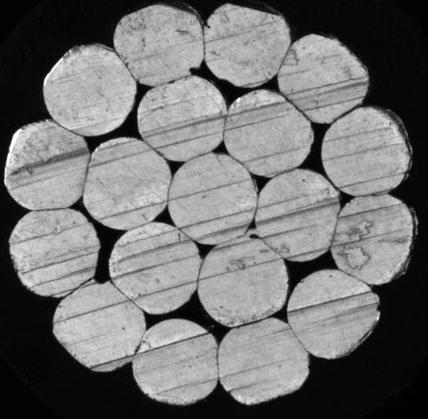

Supplement: S1 Data — (ZIP) [file pone.0300260.s001.zip › Paper_data/Fig_3/2_column/4.jpg]

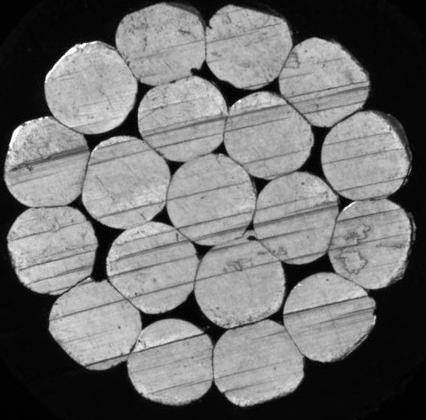

Supplement: S1 Data — (ZIP) [file pone.0300260.s001.zip › Paper_data/Fig_3/2_column/5.jpg]

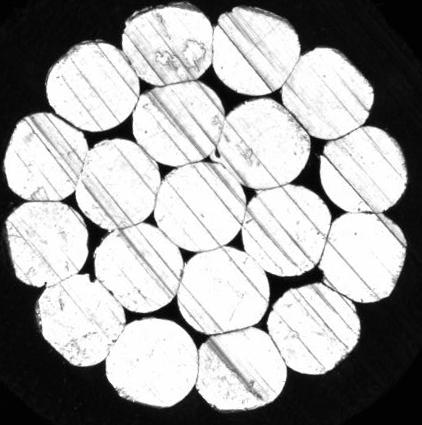

Supplement: S1 Data — (ZIP) [file pone.0300260.s001.zip › Paper_data/Fig_3/2_column/6.jpg]

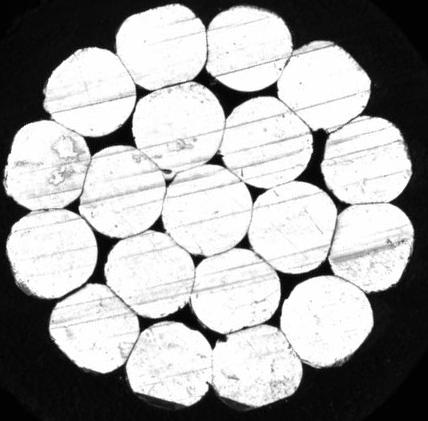

Supplement: S1 Data — (ZIP) [file pone.0300260.s001.zip › Paper_data/Fig_3/2_column/7.jpg]

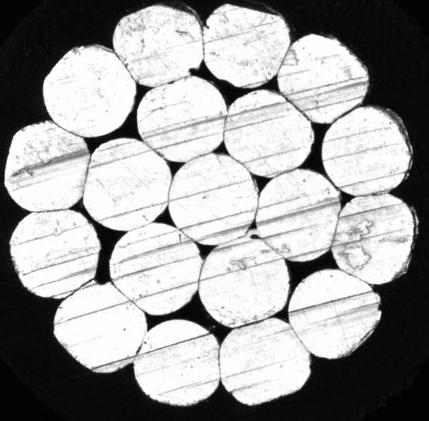

Supplement: S1 Data — (ZIP) [file pone.0300260.s001.zip › Paper_data/Fig_3/2_column/8.jpg]

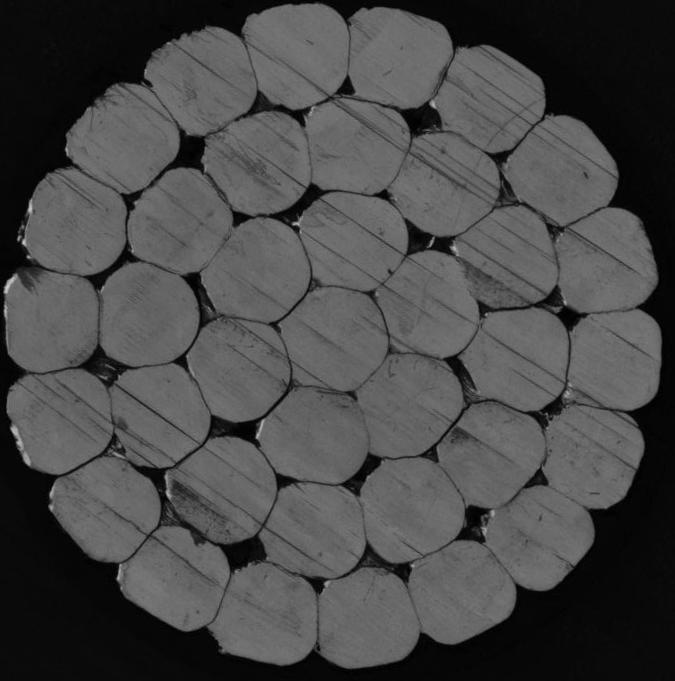

Supplement: S1 Data — (ZIP) [file pone.0300260.s001.zip › Paper_data/Fig_3/3_column/1.jpg]

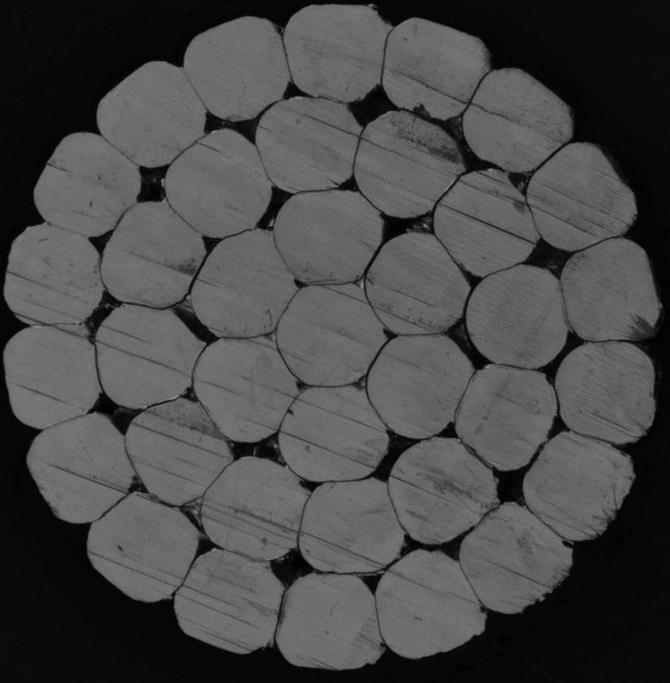

Supplement: S1 Data — (ZIP) [file pone.0300260.s001.zip › Paper_data/Fig_3/3_column/2.jpg]

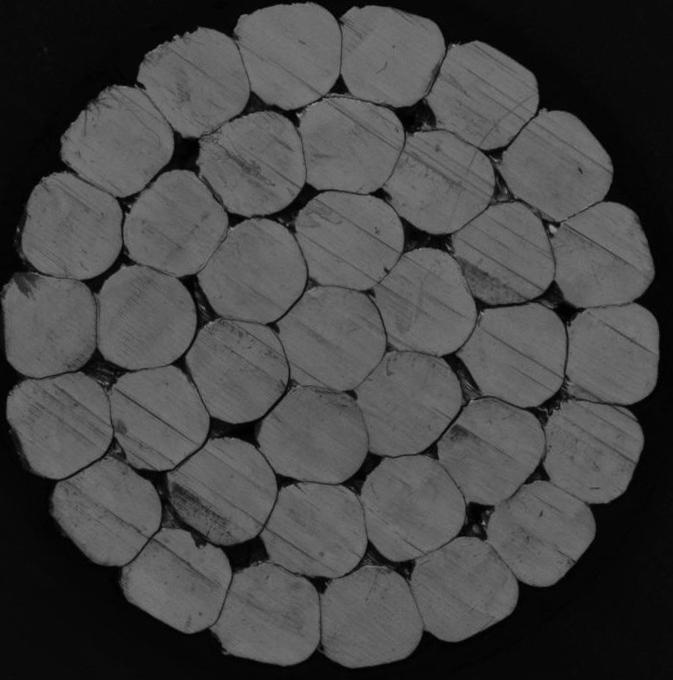

Supplement: S1 Data — (ZIP) [file pone.0300260.s001.zip › Paper_data/Fig_3/3_column/3.jpg]

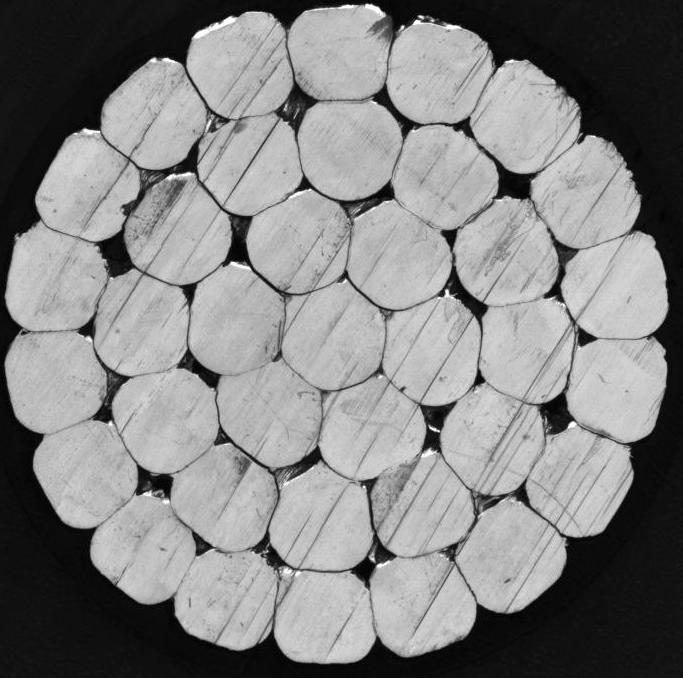

Supplement: S1 Data — (ZIP) [file pone.0300260.s001.zip › Paper_data/Fig_3/3_column/4.jpg]

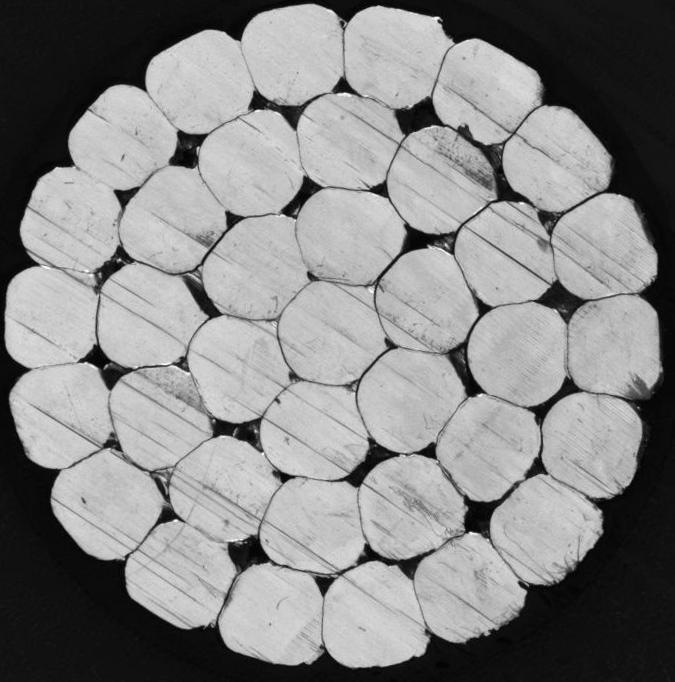

Supplement: S1 Data — (ZIP) [file pone.0300260.s001.zip › Paper_data/Fig_3/3_column/5.jpg]

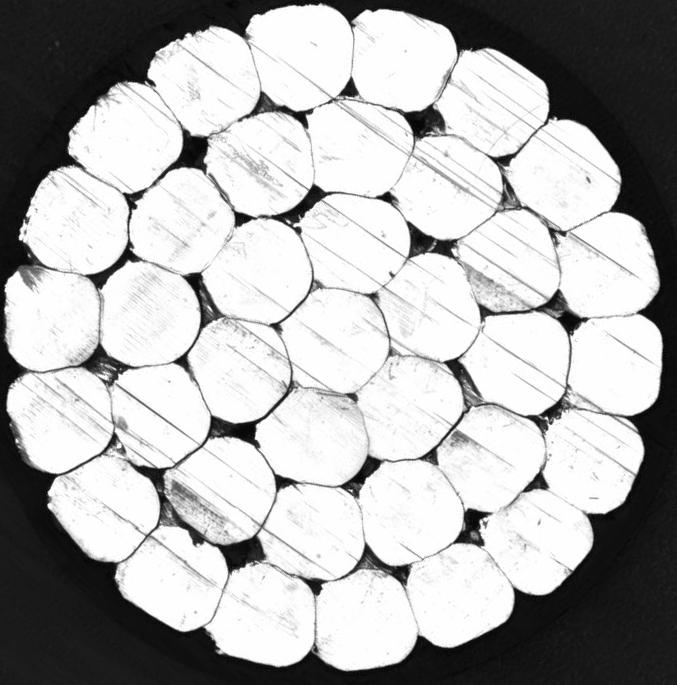

Supplement: S1 Data — (ZIP) [file pone.0300260.s001.zip › Paper_data/Fig_3/3_column/6.jpg]

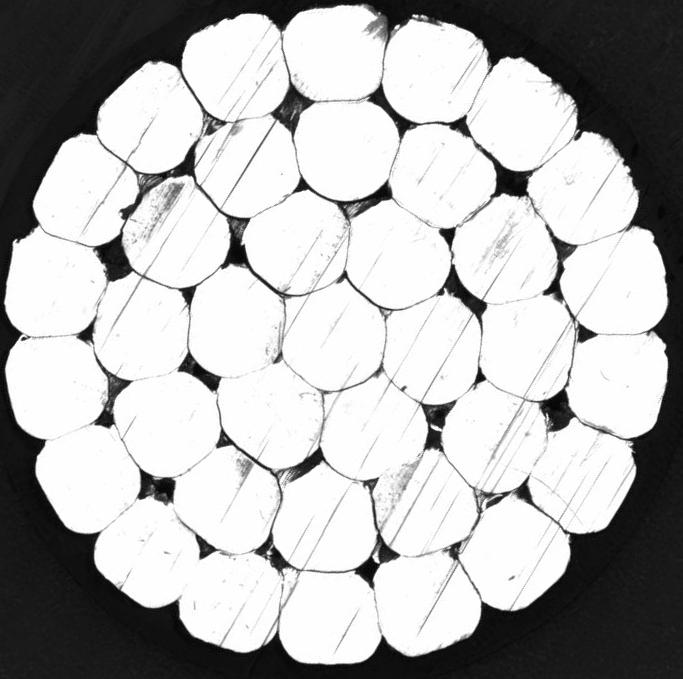

Supplement: S1 Data — (ZIP) [file pone.0300260.s001.zip › Paper_data/Fig_3/3_column/7.jpg]

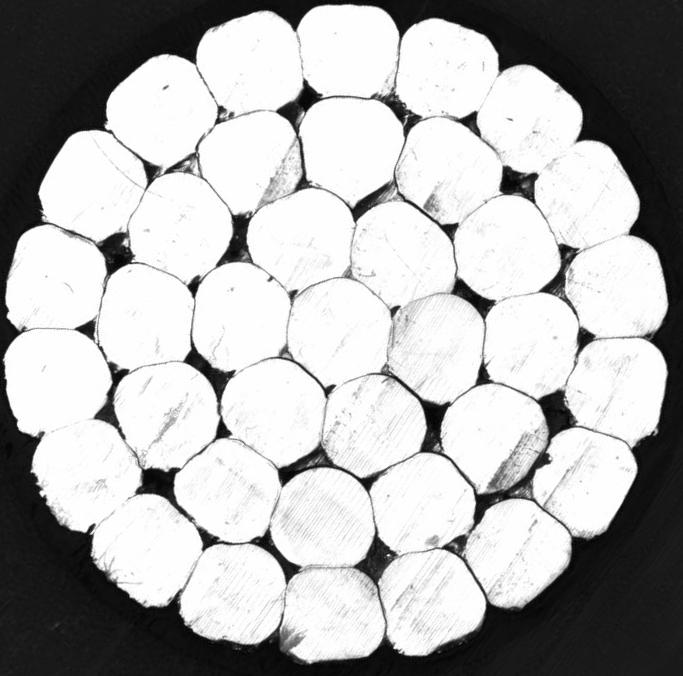

Supplement: S1 Data — (ZIP) [file pone.0300260.s001.zip › Paper_data/Fig_3/3_column/8.jpg]

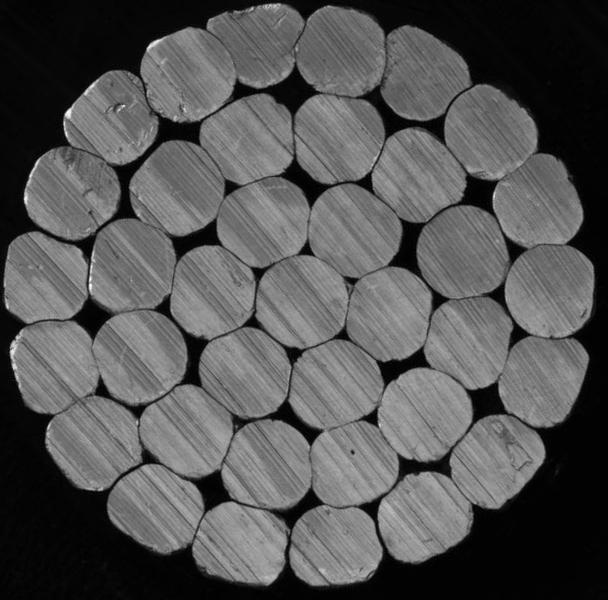

Supplement: S1 Data — (ZIP) [file pone.0300260.s001.zip › Paper_data/Fig_3/4_column/1.jpg]

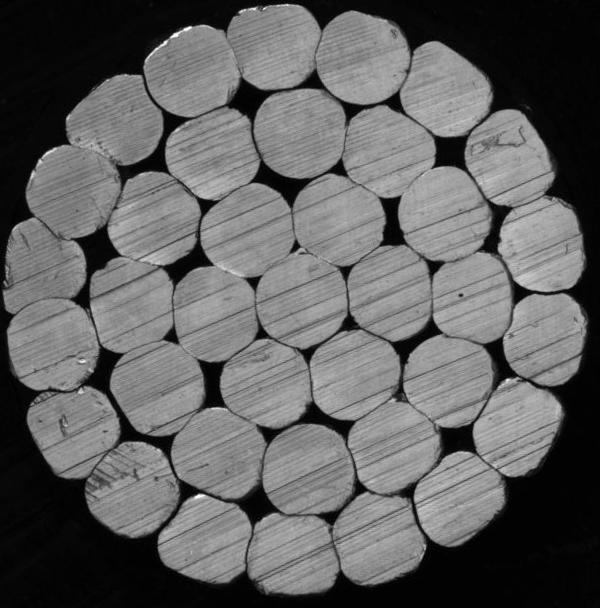

Supplement: S1 Data — (ZIP) [file pone.0300260.s001.zip › Paper_data/Fig_3/4_column/2.jpg]

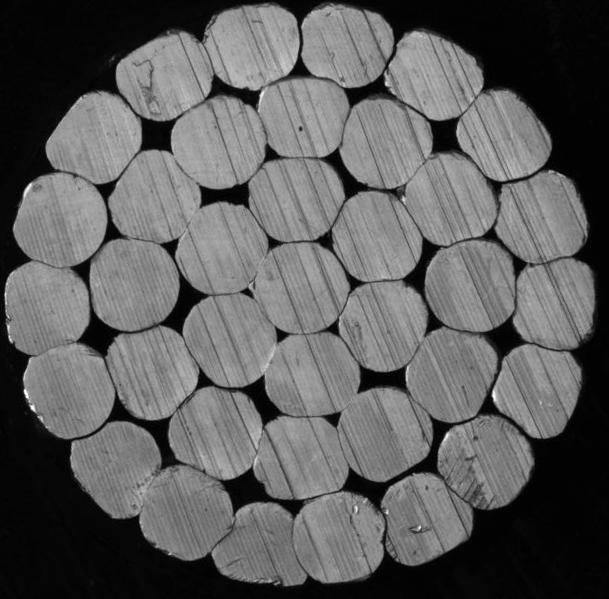

Supplement: S1 Data — (ZIP) [file pone.0300260.s001.zip › Paper_data/Fig_3/4_column/3.jpg]

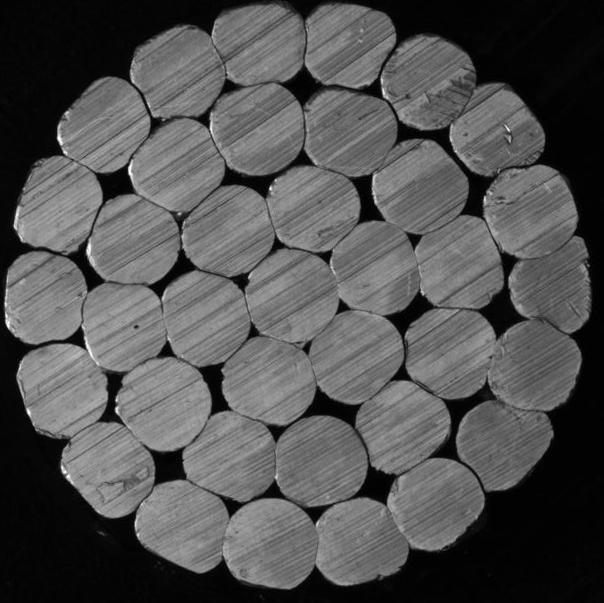

Supplement: S1 Data — (ZIP) [file pone.0300260.s001.zip › Paper_data/Fig_3/4_column/4.jpg]

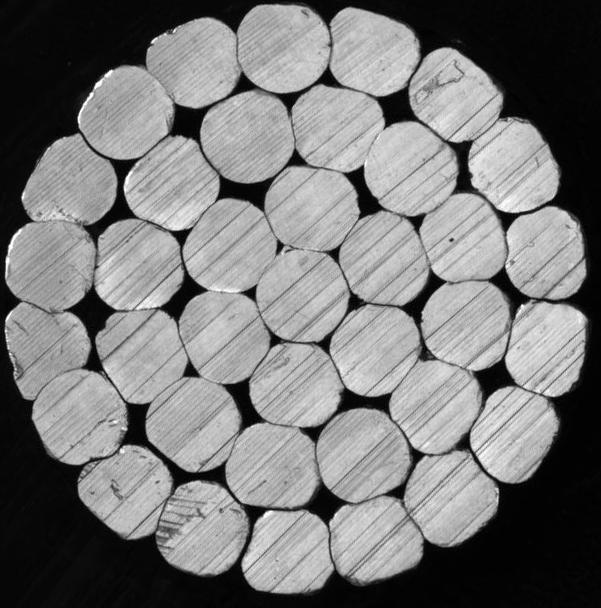

Supplement: S1 Data — (ZIP) [file pone.0300260.s001.zip › Paper_data/Fig_3/4_column/5.jpg]

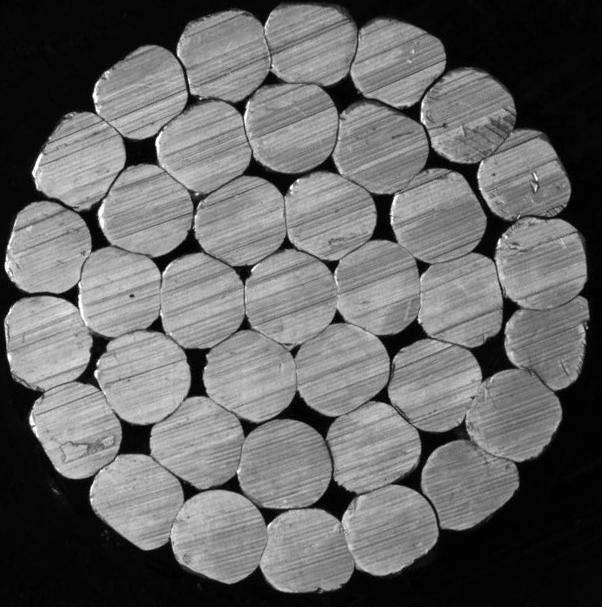

Supplement: S1 Data — (ZIP) [file pone.0300260.s001.zip › Paper_data/Fig_3/4_column/6.jpg]

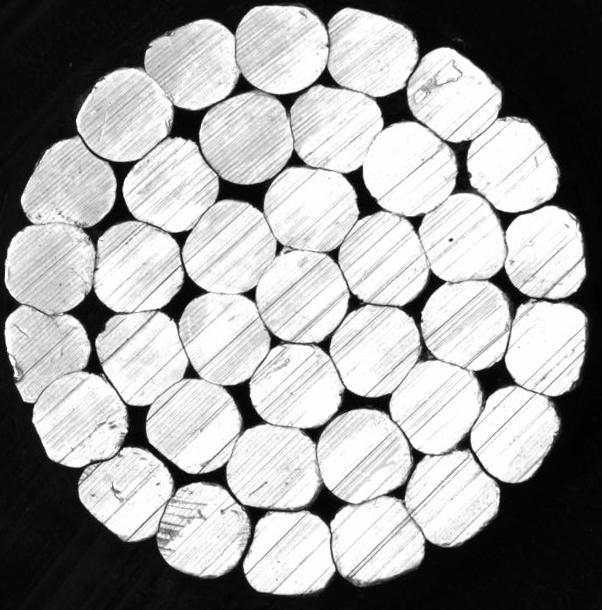

Supplement: S1 Data — (ZIP) [file pone.0300260.s001.zip › Paper_data/Fig_3/4_column/7.jpg]

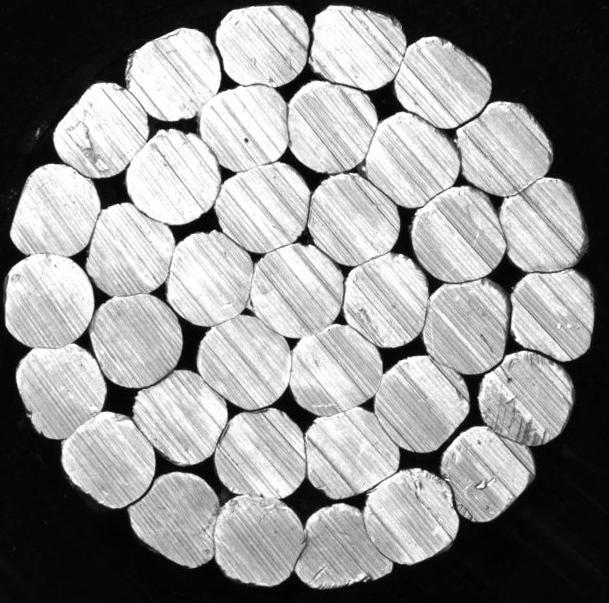

Supplement: S1 Data — (ZIP) [file pone.0300260.s001.zip › Paper_data/Fig_3/4_column/8.jpg]

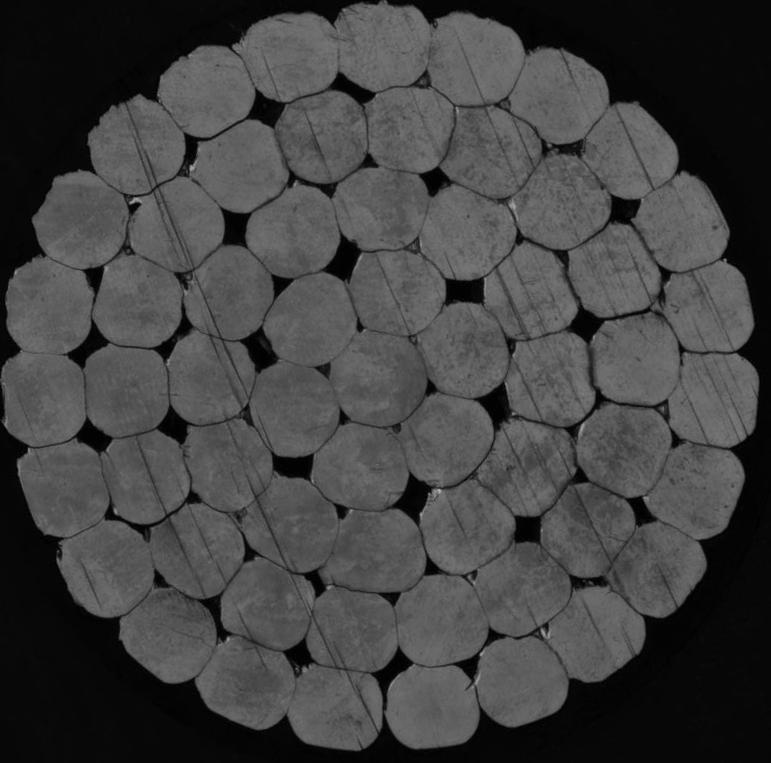

Supplement: S1 Data — (ZIP) [file pone.0300260.s001.zip › Paper_data/Fig_3/5_column/1.jpg]

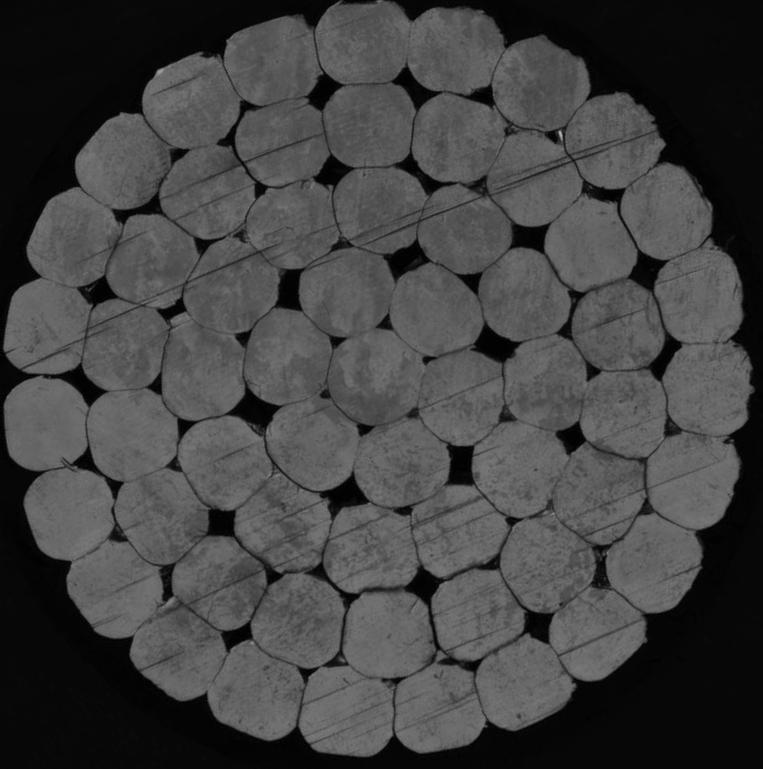

Supplement: S1 Data — (ZIP) [file pone.0300260.s001.zip › Paper_data/Fig_3/5_column/2.jpg]

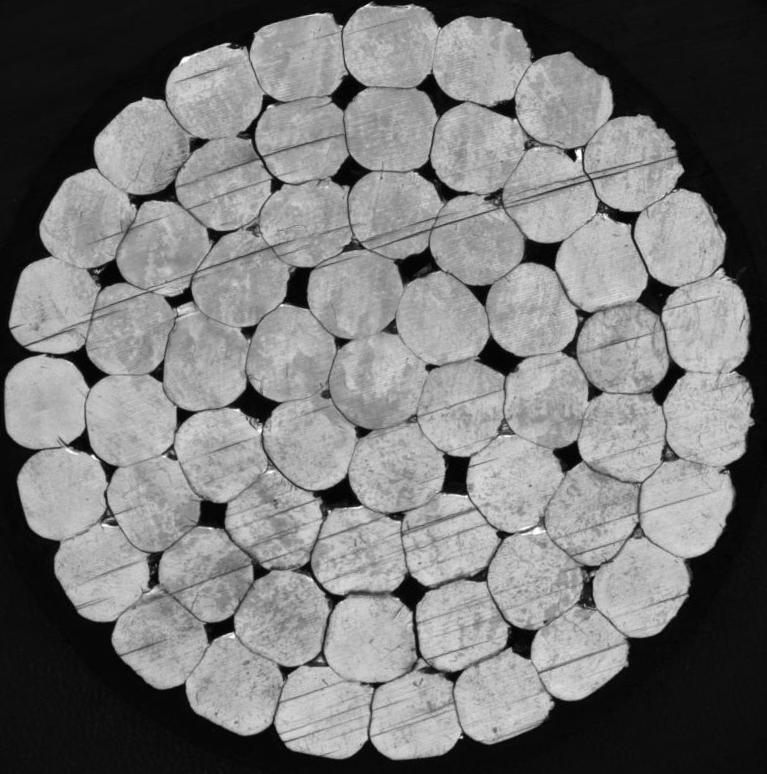

Supplement: S1 Data — (ZIP) [file pone.0300260.s001.zip › Paper_data/Fig_3/5_column/3.jpg]

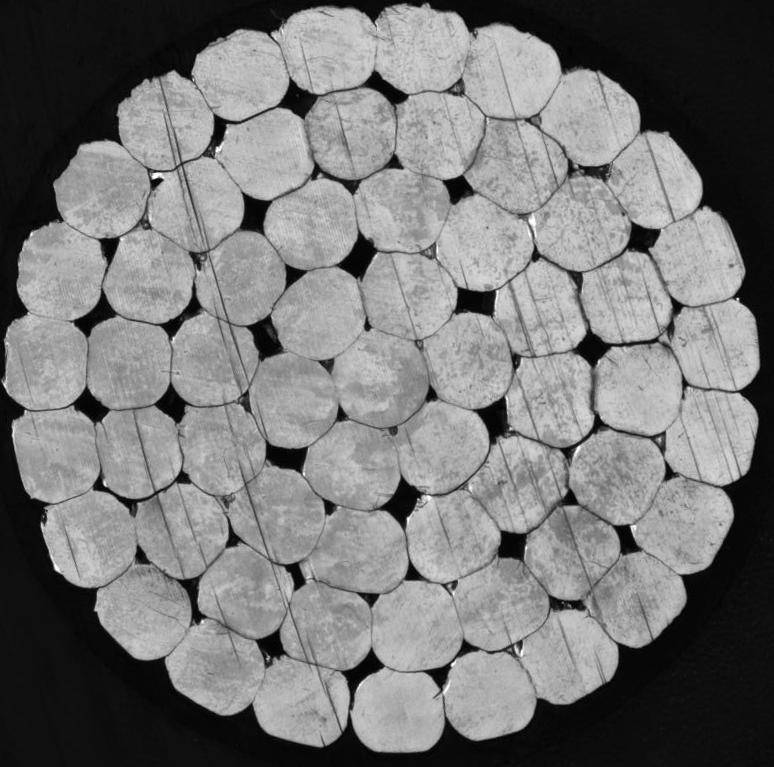

Supplement: S1 Data — (ZIP) [file pone.0300260.s001.zip › Paper_data/Fig_3/5_column/4.jpg]

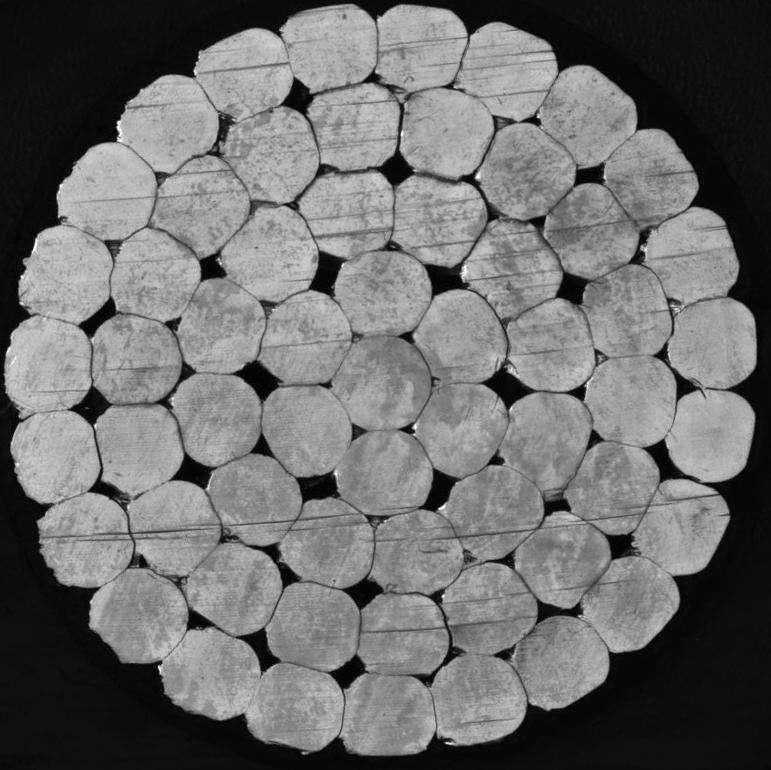

Supplement: S1 Data — (ZIP) [file pone.0300260.s001.zip › Paper_data/Fig_3/5_column/5.jpg]

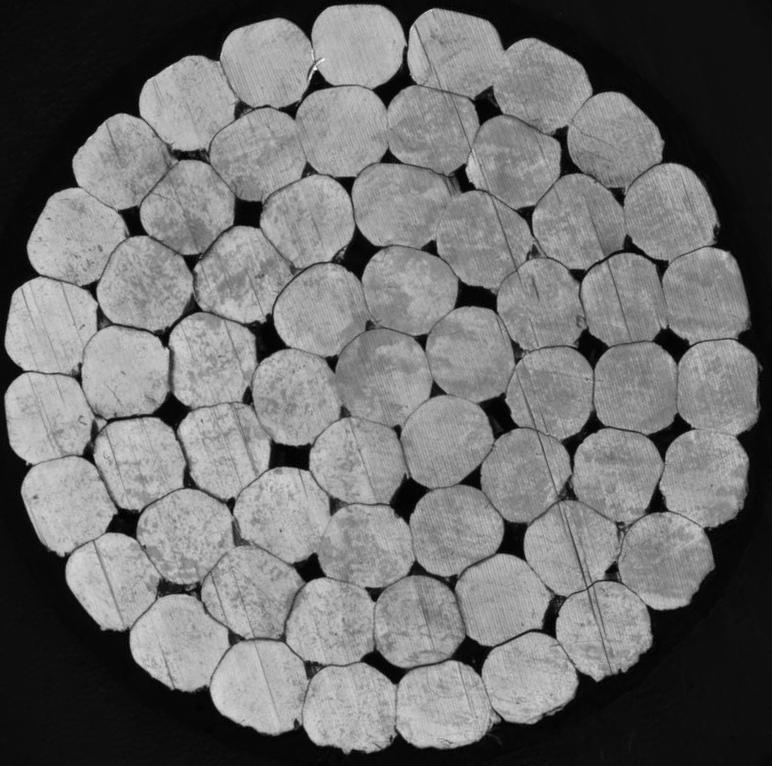

Supplement: S1 Data — (ZIP) [file pone.0300260.s001.zip › Paper_data/Fig_3/5_column/6.jpg]

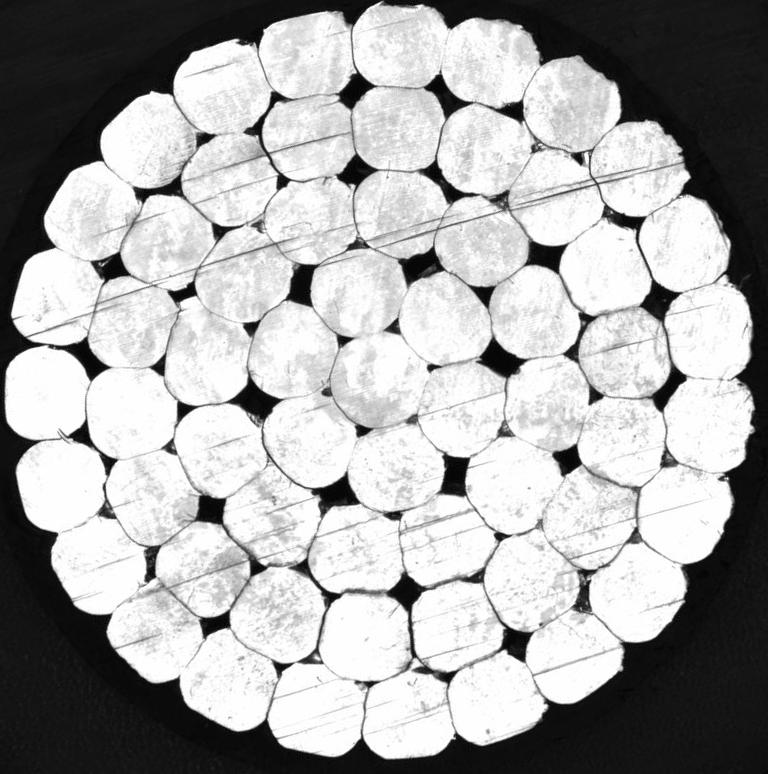

Supplement: S1 Data — (ZIP) [file pone.0300260.s001.zip › Paper_data/Fig_3/5_column/7.jpg]

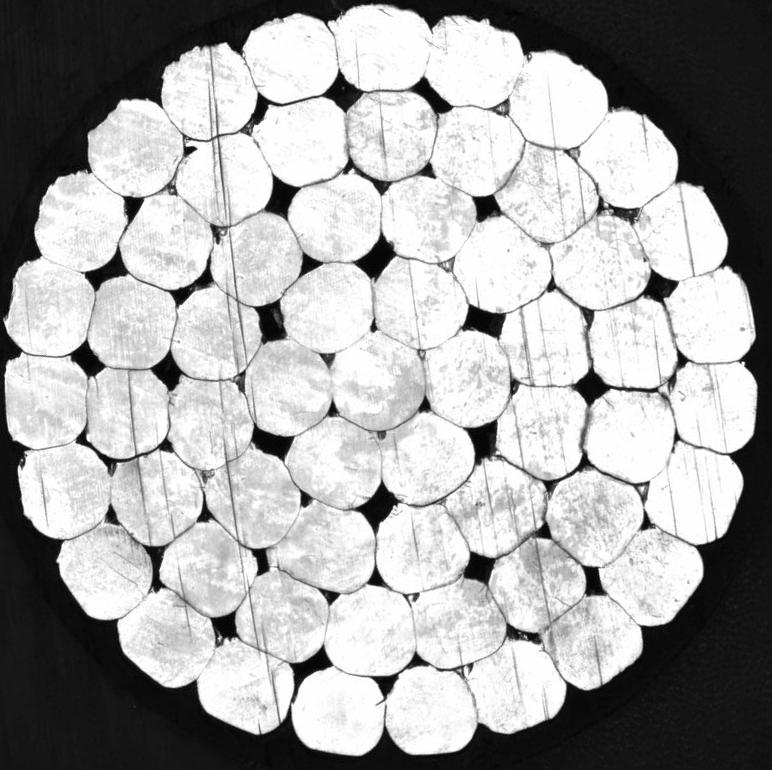

Supplement: S1 Data — (ZIP) [file pone.0300260.s001.zip › Paper_data/Fig_3/5_column/8.jpg]

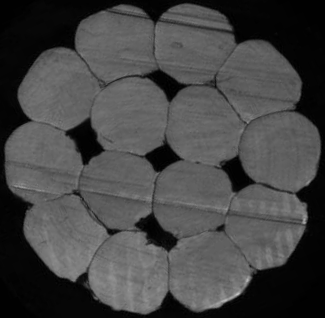

Supplement: S1 Data — (ZIP) [file pone.0300260.s001.zip › Paper_data/Fig_4/a/1.png]

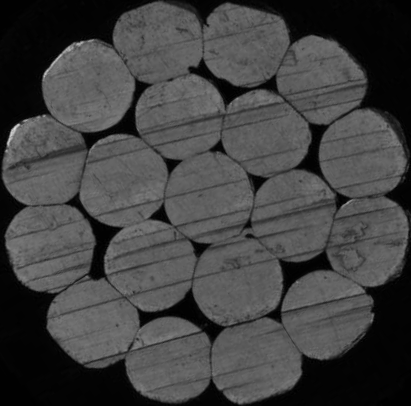

Supplement: S1 Data — (ZIP) [file pone.0300260.s001.zip › Paper_data/Fig_4/a/2.png]

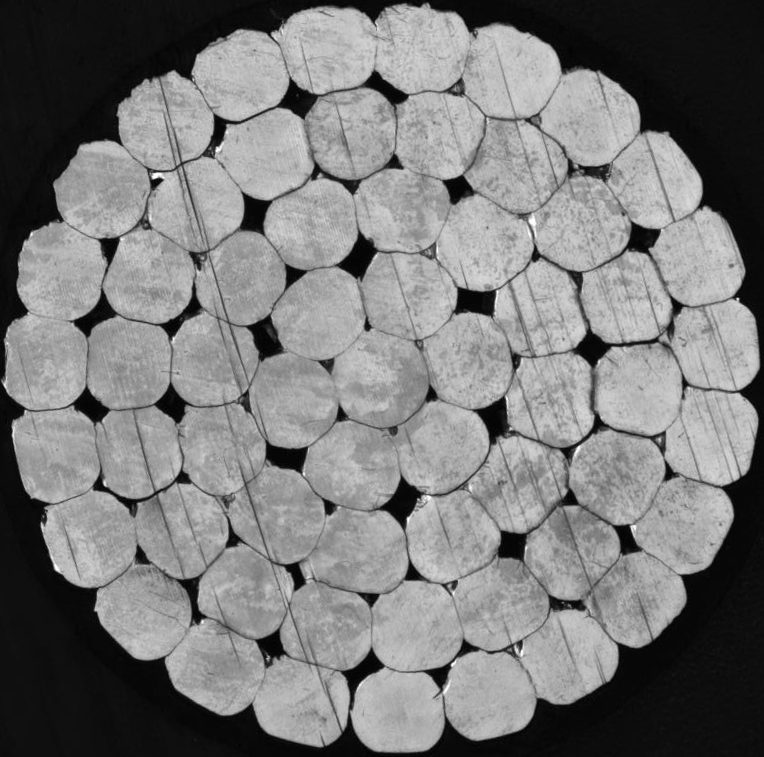

Supplement: S1 Data — (ZIP) [file pone.0300260.s001.zip › Paper_data/Fig_4/a/5.png]

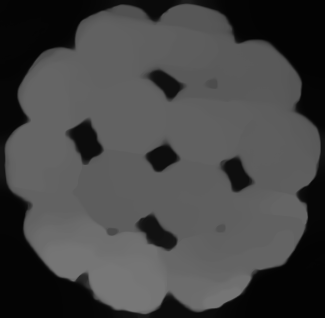

Supplement: S1 Data — (ZIP) [file pone.0300260.s001.zip › Paper_data/Fig_4/b/1.png]

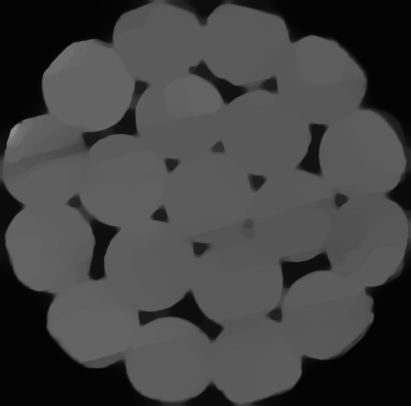

Supplement: S1 Data — (ZIP) [file pone.0300260.s001.zip › Paper_data/Fig_4/b/2.png]

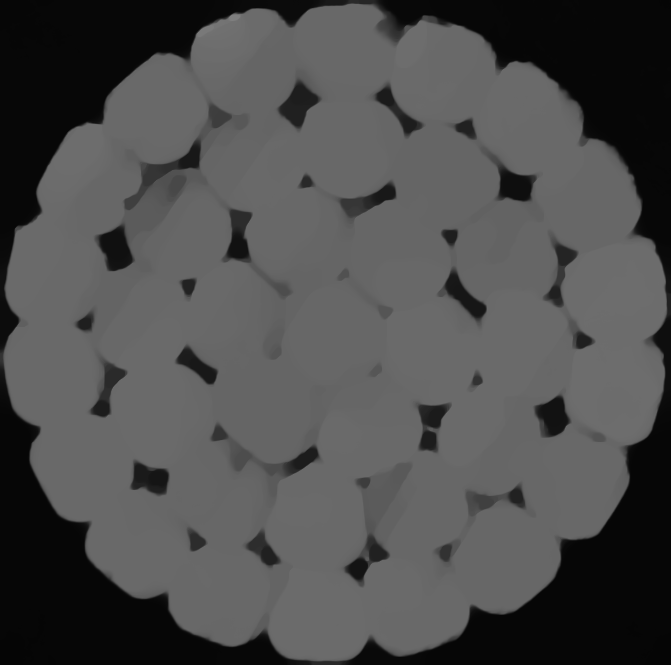

Supplement: S1 Data — (ZIP) [file pone.0300260.s001.zip › Paper_data/Fig_4/b/3.png]

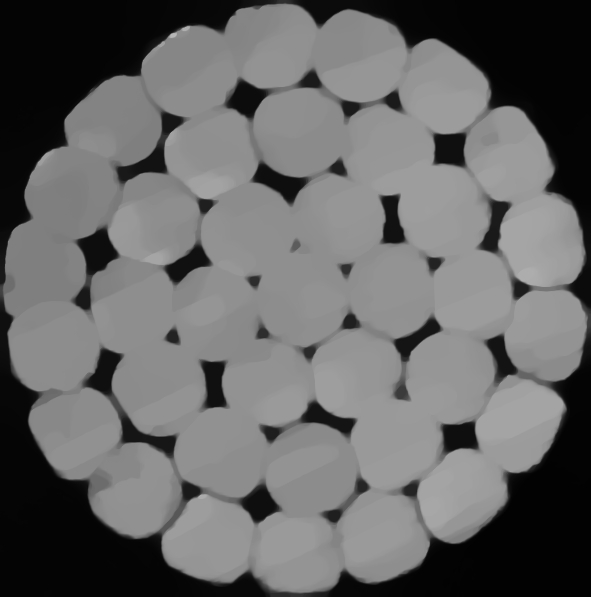

Supplement: S1 Data — (ZIP) [file pone.0300260.s001.zip › Paper_data/Fig_4/b/4.png]

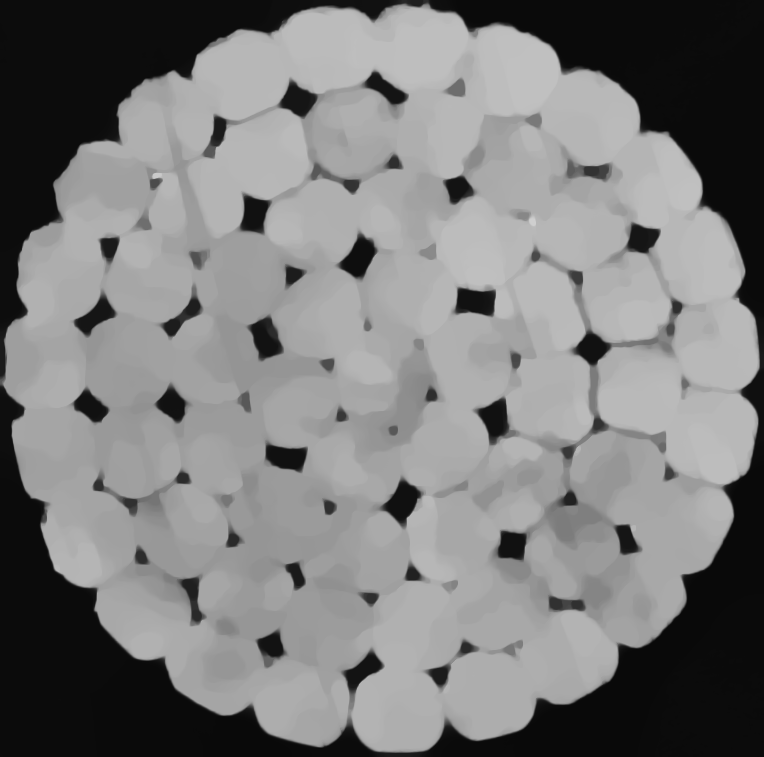

Supplement: S1 Data — (ZIP) [file pone.0300260.s001.zip › Paper_data/Fig_4/b/5.png]

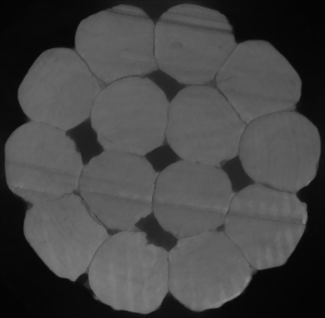

Supplement: S1 Data — (ZIP) [file pone.0300260.s001.zip › Paper_data/Fig_4/c/1.png]

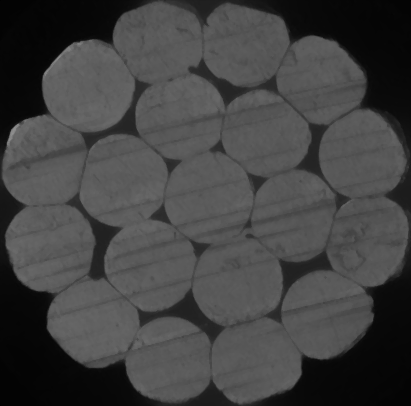

Supplement: S1 Data — (ZIP) [file pone.0300260.s001.zip › Paper_data/Fig_4/c/2.png]

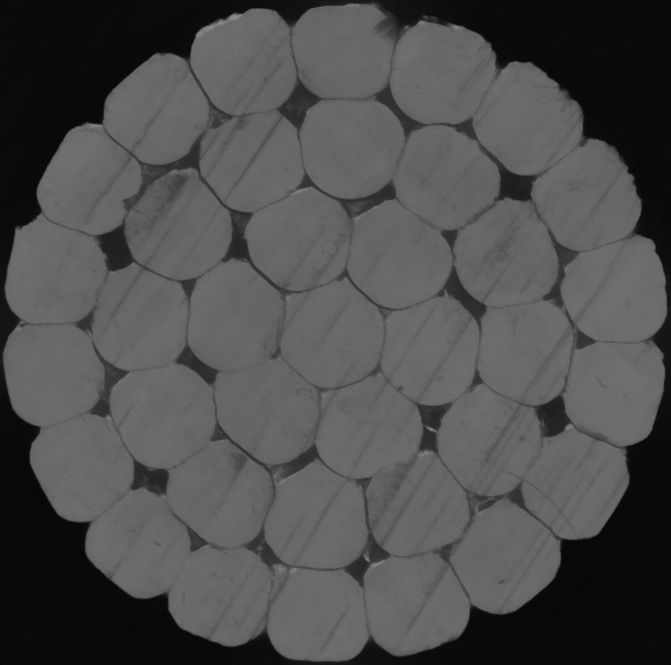

Supplement: S1 Data — (ZIP) [file pone.0300260.s001.zip › Paper_data/Fig_4/c/3.png]

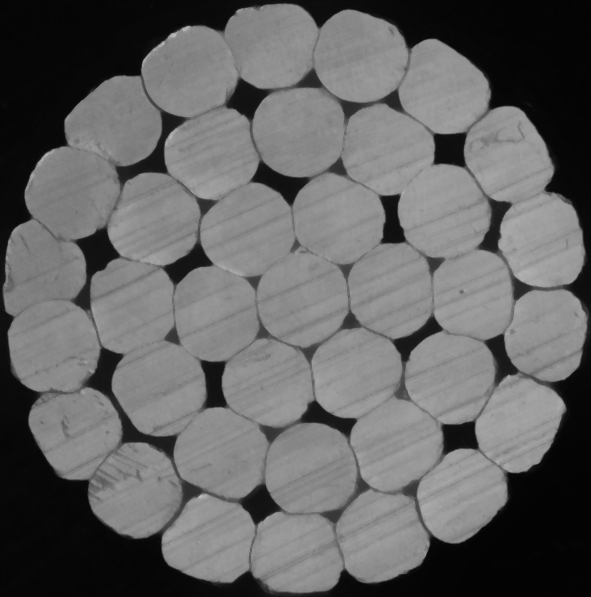

Supplement: S1 Data — (ZIP) [file pone.0300260.s001.zip › Paper_data/Fig_4/c/4.png]

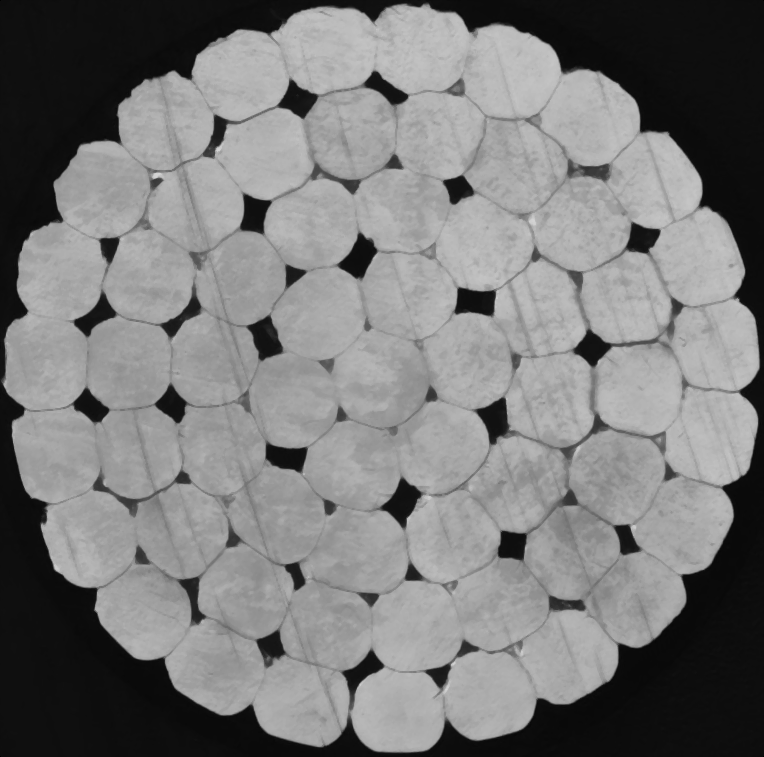

Supplement: S1 Data — (ZIP) [file pone.0300260.s001.zip › Paper_data/Fig_4/c/5.png]

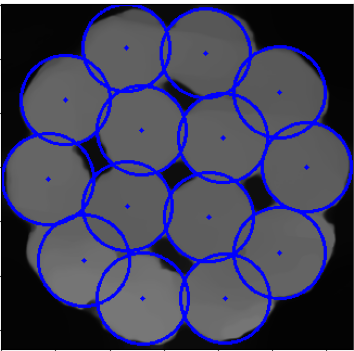

Supplement: S1 Data — (ZIP) [file pone.0300260.s001.zip › Paper_data/Fig_5/a/1.png]

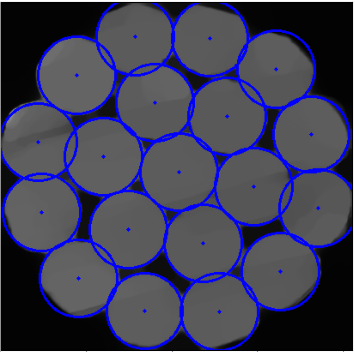

Supplement: S1 Data — (ZIP) [file pone.0300260.s001.zip › Paper_data/Fig_5/a/2.png]

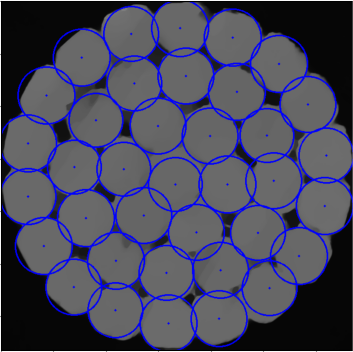

Supplement: S1 Data — (ZIP) [file pone.0300260.s001.zip › Paper_data/Fig_5/a/3.png]

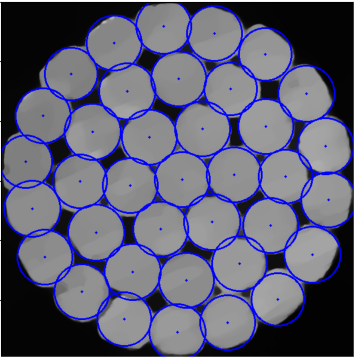

Supplement: S1 Data — (ZIP) [file pone.0300260.s001.zip › Paper_data/Fig_5/a/4.png]

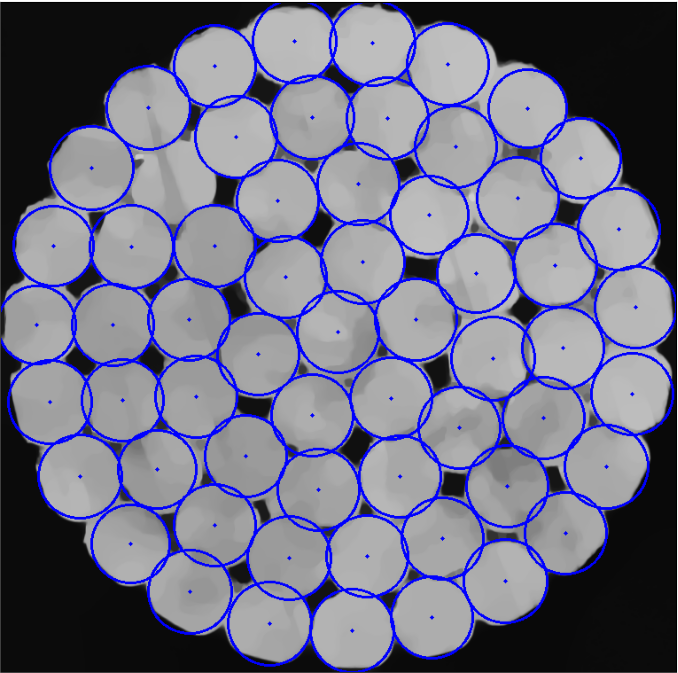

Supplement: S1 Data — (ZIP) [file pone.0300260.s001.zip › Paper_data/Fig_5/a/5.png]

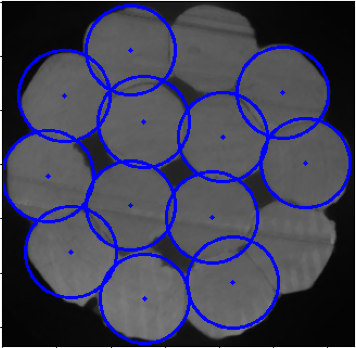

Supplement: S1 Data — (ZIP) [file pone.0300260.s001.zip › Paper_data/Fig_5/b/1.png]

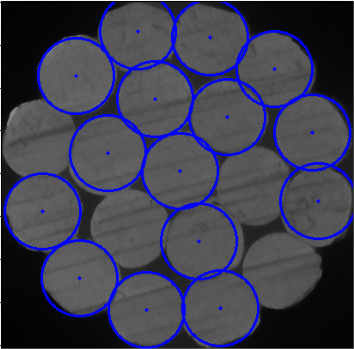

Supplement: S1 Data — (ZIP) [file pone.0300260.s001.zip › Paper_data/Fig_5/b/2.png]

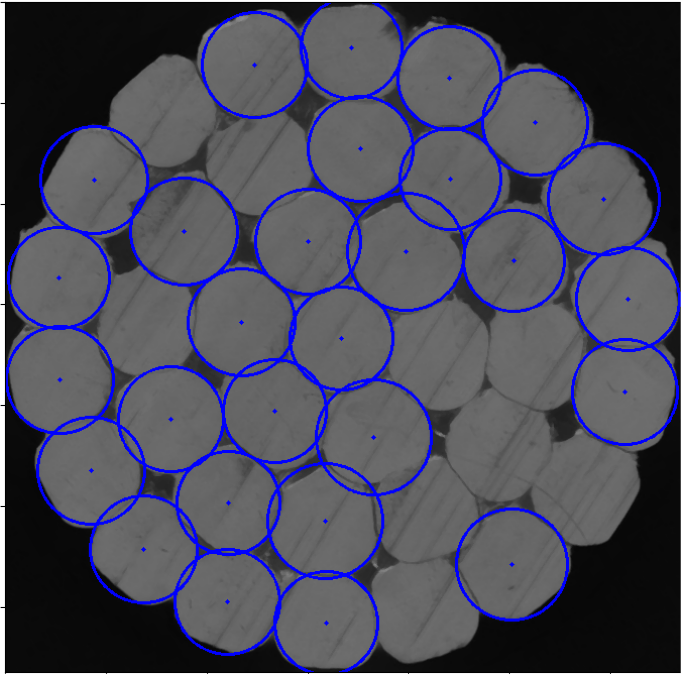

Supplement: S1 Data — (ZIP) [file pone.0300260.s001.zip › Paper_data/Fig_5/b/3.png]

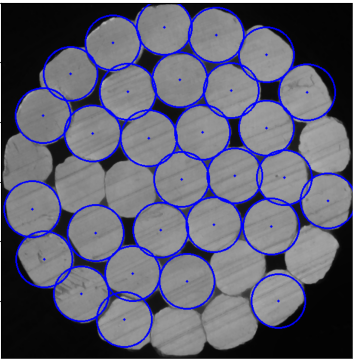

Supplement: S1 Data — (ZIP) [file pone.0300260.s001.zip › Paper_data/Fig_5/b/4.png]

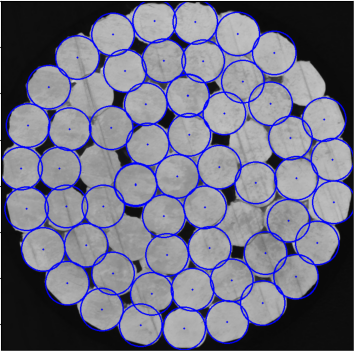

Supplement: S1 Data — (ZIP) [file pone.0300260.s001.zip › Paper_data/Fig_5/b/5.png]

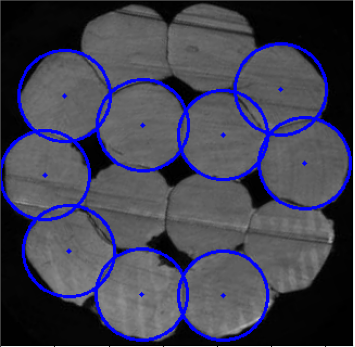

Supplement: S1 Data — (ZIP) [file pone.0300260.s001.zip › Paper_data/Fig_5/c/1.png]

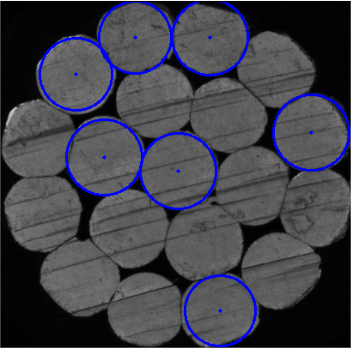

Supplement: S1 Data — (ZIP) [file pone.0300260.s001.zip › Paper_data/Fig_5/c/2.png]

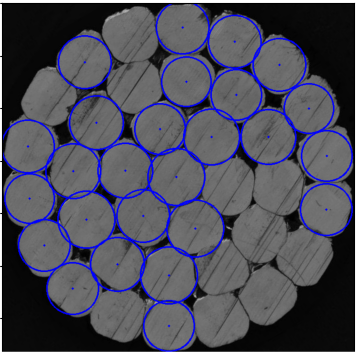

Supplement: S1 Data — (ZIP) [file pone.0300260.s001.zip › Paper_data/Fig_5/c/3.png]

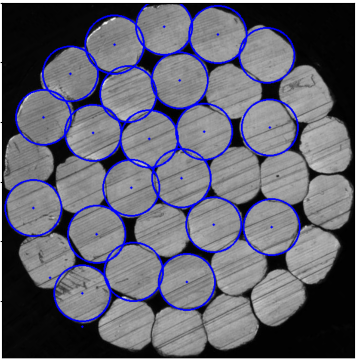

Supplement: S1 Data — (ZIP) [file pone.0300260.s001.zip › Paper_data/Fig_5/c/4.png]

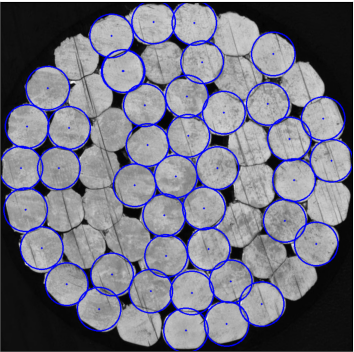

Supplement: S1 Data — (ZIP) [file pone.0300260.s001.zip › Paper_data/Fig_5/c/5.png]

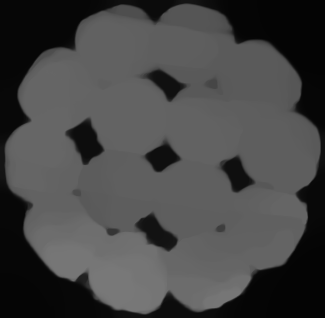

Supplement: S1 Data — (ZIP) [file pone.0300260.s001.zip › Paper_data/Fig_6/c/1.png]

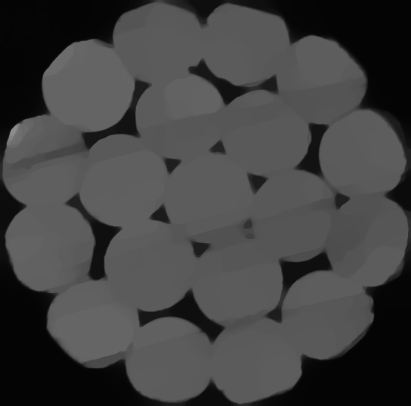

Supplement: S1 Data — (ZIP) [file pone.0300260.s001.zip › Paper_data/Fig_6/c/2.png]

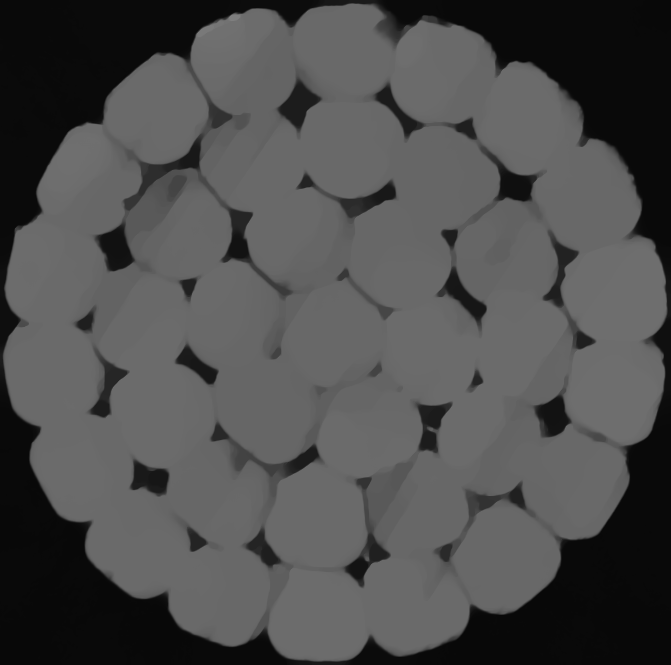

Supplement: S1 Data — (ZIP) [file pone.0300260.s001.zip › Paper_data/Fig_6/c/3.png]

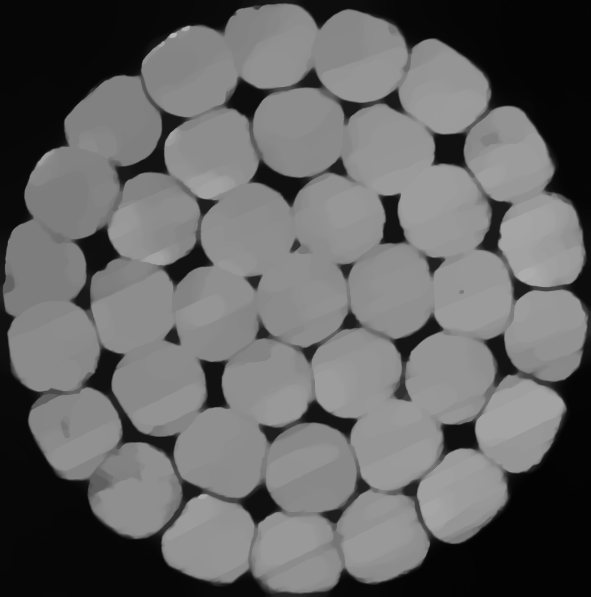

Supplement: S1 Data — (ZIP) [file pone.0300260.s001.zip › Paper_data/Fig_6/c/4.png]

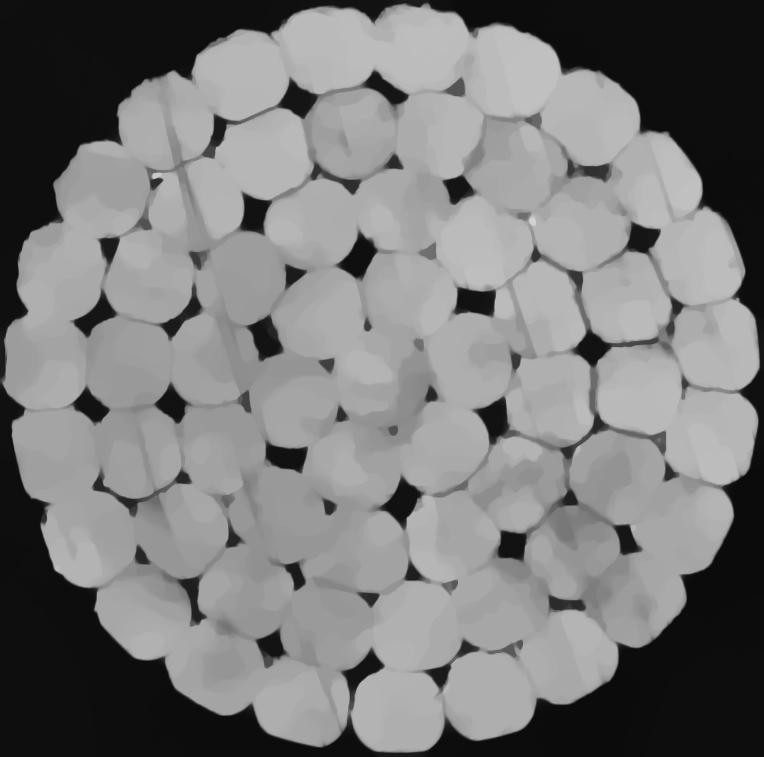

Supplement: S1 Data — (ZIP) [file pone.0300260.s001.zip › Paper_data/Fig_6/c/5.png]

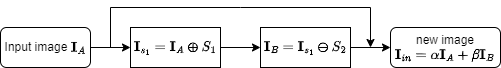

Supplement: S1 Data — (ZIP) [file pone.0300260.s001.zip › Paper_data/flowchart.png]
